# aPKC-ζ III promotes trophoblast fusion by altering Par-3 interactions with Hippo signaling kinase LATS1

**DOI:** 10.1016/j.stemcr.2026.102975

**Published:** 2026-06-25

**Authors:** Sumaiyah Z. Shaha, Wendy K. Duan, Juan Garcia Rivas, Ivan K. Domingo, Meghan Riddell

**Affiliations:** 1 Department of Physiology, University of Alberta, Edmonton, AB, Canada; 2 Department of Obstetrics and Gynecology, University of Alberta, Edmonton, AB, Canada

**Keywords:** Trophoblast, aPKC, single-nuclei RNA sequencing, trophoblast stem cells, Hippo, placenta

## Abstract

The first trimester of pregnancy is a critical developmental period for the placenta. In humans, the maternal-facing exchange surface is formed by a single giant multinucleate syncytium: the syncytiotrophoblast (ST). The ST arises from villous lineage commitment of trophoblast stem cells (TSC) and the differentiation and fusion of progenitor cytotrophoblasts (pCT) to form the multinucleate syncytium. The Hippo signaling co-transcription factor YAP1 promotes pCT maintenance and TSC stemness; however, how Hippo signaling is regulated remains unknown. We have identified a novel *PRKCZ*-encoded aPKC isoform, aPKC-ζ III, that is highly expressed in pCT and ST. Here, we establish that aPKC-ζ III promotes pCT fusion by activation of Hippo signaling. Specifically, aPKC-ζ III outcompetes the Hippo kinase LATS1 for scaffolding protein Par-3 binding, resulting in YAP1 inactivation and pCT fusion. Our findings identify a key modulator of Hippo signaling in human trophoblasts that is critical for first-trimester ST differentiation.

## Highlights

• A novel aPKC-ζ isoform expressed in the human placenta, aPKC-ζ III, promotes trophoblast fusion
• aPKC-ζ III is significantly upregulated during trophoblast fusion
• We identified aPKC-ζ III as an upstream activator of the Hippo signaling pathway in the human placenta

## Graphical abstract

**Figure:**
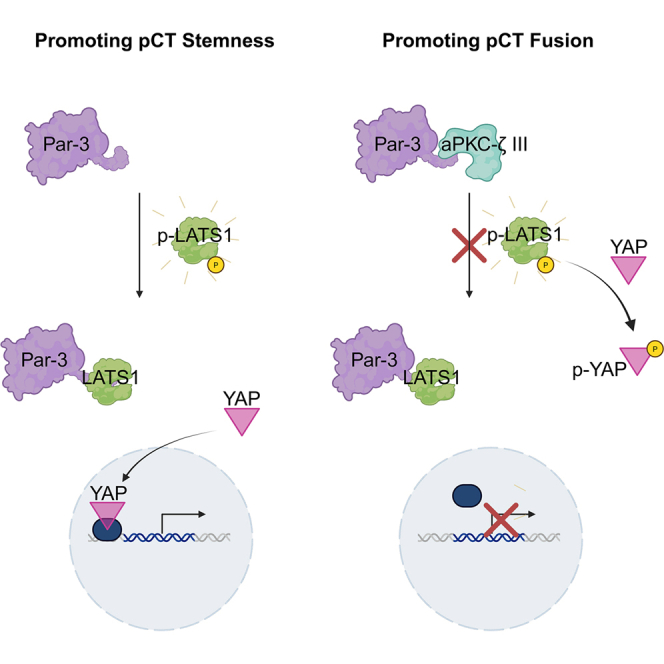

## Teaser

In this article, Riddell and colleagues identify that a novel human placental-specific *PRKCZ* isoform, aPKC-ζ III, promotes progenitor cytotrophoblast fusion during first-trimester development. Mechanistically, aPKC-ζ III outcompetes the Hippo kinase LATS1 for binding to the scaffolding protein Par-3. This molecular swap activates Hippo signaling and inactivates the stemness factor YAP1, revealing a critical regulatory pathway governing early placental differentiation.

## Introduction

Formation of the placenta is critical for the establishment and progression of pregnancy. It is a fetally derived organ that is responsible for facilitating nutrient transport, gas exchange, and hormone secretion, among other critical functions. Trophoblasts are placental-specific epithelial cells. Trophoblast stem cells (TSCs) are derived from the trophectoderm (TE) of the blastocyst; thus, this represents the first committed cell lineage during development. In humans, the syncytiotrophoblast (ST) is the terminally differentiated cell of the villous trophoblast lineage. It is a giant multinucleate cell that spans the surface of the maternal-facing exchange surface of the placenta. This single giant cell facilitates the transfer of gases and nutrients, while secreting pregnancy-specific hormones to promote placental development and maternal adaptation to pregnancy (Turco and Moffett, 2019). The ST is post-mitotic and relies on the differentiation of underlying progenitor cytotrophoblasts (pCTs) for its expansion and maintenance (Mi et al., 2000). The differentiation of pCT into ST is a complex multistep process that culminates in cell-cell fusion and upregulation of genes specific for ST function and homeostasis (Gerbaud and Pidoux, 2015). In common pregnancy complications such as intrauterine growth restriction and preeclampsia, there are defects in pCT to ST fusion that are thought to arise within early placental formation (Langbein et al., 2008; Ruebner et al., 2012). Thus, understanding pCT to ST differentiation in the first trimester is critical.

The Hippo signaling pathway is involved in regulating organ growth, cell proliferation, and differentiation in many cellular contexts (Meng et al., 2016; Yu et al., 2015). Hippo signaling is regulated by numerous signals such as cell contact, mechanical cues, stress, and cell polarity (Meng et al., 2016). When Hippo signaling is active, mammalian Ste20-like kinases 1/2 (MST1/2) phosphorylate and activate large tumor suppressor 1/2 (LATS1/2), which in turn phosphorylates co-transcription factors Yes-associated protein 1 (YAP) and transcriptional coactivation for PDZ-binding motif (TAZ), resulting in YAP/TAZ cytoplasmic retention and/or ubiquitination and degradation (Chan et al., 2005; Hao et al., 2008). When Hippo signaling is disrupted, non-phosphorylated YAP/TAZ (active) translocate to the nucleus and interact with DNA-binding transcriptional enhanced associate domains 1–4 (TEAD1–4) to influence transcription (Lin et al., 2017; Vassilev et al., 2001). In human trophoblasts, Hippo signaling has been established as a critical regulator of pCT maintenance. YAP is highly expressed within the nucleus in the pCT population resulting in TEAD4 activity. This leads to the transcription of genes promoting trophoblast stemness and repression of genes necessary for cell fusion and ST differentiation (Meinhardt et al., 2020; Mizutani et al., 2022). While it is understood that Hippo signaling is critical for pCT maintenance, what regulates Hippo signaling in trophoblasts has not yet been examined.

During human TE segregation from the inner cell mass, the outer cells become polarized and YAP1 localizes to the nucleus. This initiation and maintenance of cell polarity is governed by the activity of an apical-basal polarity regulator, atypical protein kinase C (aPKC). When TE aPKC expression or activity is disrupted, YAP1 localization is altered (Gerri et al., 2020). Therefore, in the pre-TSC TE, Hippo signaling and cell polarity are linked. Cell polarity regulatory complexes are well known to play important roles in epithelial cell maintenance and differentiation; however, studies examining polarity regulators in the human placenta are limited (Shaha et al., 2023; Wen and Zhang, 2018). The Par complex is an evolutionarily conserved polarity-regulating complex that consists of scaffolding proteins partitioning defective-3 (Par-3) and partitioning defective-6 (Par-6) and aPKC isoforms. There are two main isoforms of aPKC in humans: aPKC-ɩ and aPKC-ζ encoded by *PRKCI* and *PRKCZ*, respectively. aPKCs are spatio-temporally regulated as their full kinase activation is dependent upon protein-protein interactions (Graybill et al., 2012). Murine models have revealed that *Prkci* knockout (KO) is embryonic lethal by day 9 due to defects in placental development, but *Prkcz* KO are grossly normal with impairments in NF-κB signaling (Bhattacharya et al., 2020; Leitges et al., 2001; Soloff et al., 2004). Par complex members have also been implicated in human TSC and pCT to ST differentiation. Sivasubramaniyam et al. established that Par-6 negatively regulates trophoblast fusion (Sivasubramaniyam et al., 2013)^.^ Among some of the first studies performed using human TSC, aPKC-ɩ was shown to promote TSC to ST differentiation (Bhattacharya et al., 2020). Adding complexity to the canonical Par complex, we recently identified that the human placenta expresses three isoforms of aPKC: aPKC-ɩ, aPKC-ζ, and *PRKCZ*-encoded aPKC-ζ III (Shaha et al., 2022). aPKC-ζ III has an N-terminal truncation that results in a loss of the Phox and Bem1 (PB1) domain, but retained expression of the kinase domain, the pseudosubstrate inhibitory region, and the PDZ-binding motifs necessary for interaction with Par-3 (Holly et al., 2020; Shaha et al., 2022). The PB1 domain of aPKCs are necessary for interaction with Par-6 via PB1-PB1-mediated interactions. PB1 heterodimerization between aPKC and Par-6 allows for full activation of kinase activity by removal of the pseudosubstrate region from the kinase domain and coupling the activity to the plasma membrane (Dong et al., 2020; Graybill et al., 2012). Thus, it is unclear if aPKC-ζ ΙΙΙ is capable of full catalytic activity (Graybill et al., 2012). Presently, the function of aPKC-ζ III in trophoblasts is unknown.

Here, we show that aPKC-ζ III promotes pCT fusion in first-trimester trophoblasts. We identify that aPKC-ζ III interacts with Par-3 to maintain activation of Hippo signaling kinase LATS1 and inactivity of the co-transcription factor YAP1 to promote pCT fusion.

## Results

### *PRKCZ* is upregulated in the villous trophoblast lineage

**Figure 1:**
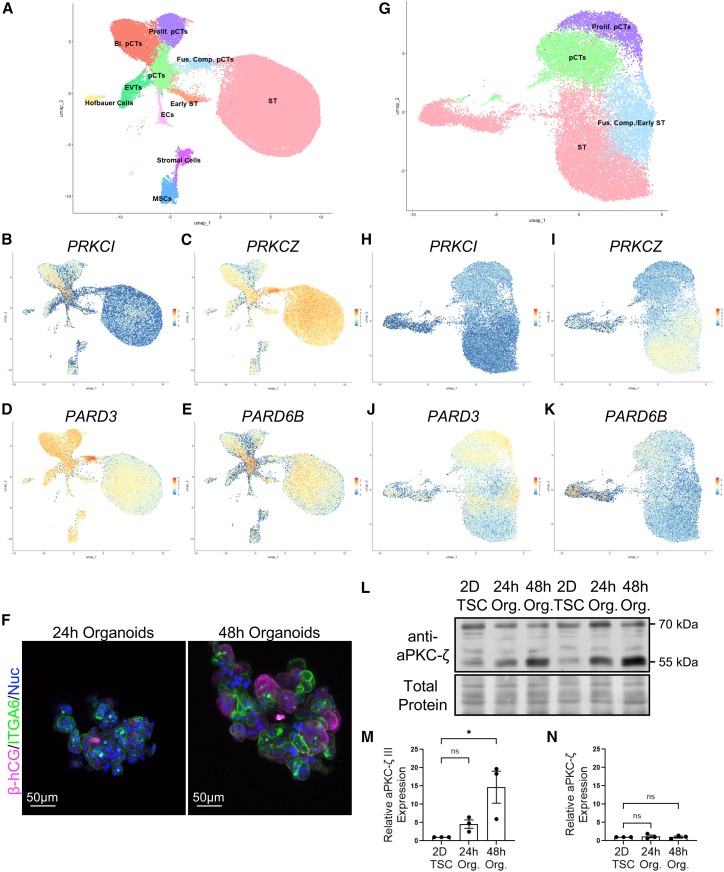
*PRKCZ*-encoded aPKC isoforms have increased expression in the villous lineage (A) UMAP of nuclei from early first-trimester placenta; *n* = 45,697 nuclei. (B–E) Dimensional reduction plots of (B) *PRKCI*, (C) *PRKCZ*, (D) *PARD3*, and (E) *PARD6B*. (F) Representative Z-projection images of 24- and 48-h ST organoids stained for ITGA6 (green), β-hCG (magenta), and nuclei (blue). (G) UMAP of nuclei from 48-h human trophoblast organoids; *n* = 22,250 nuclei. (H–K) Dimensional reduction plots of (H) *PRKCI*, (I) *PRKCZ*, (J) *PARD3*, and (K) *PARD6B*. (L) Representative western blot of aPKC-ζ and aPKC-ζ III in 2D TSCs and 24- and 48-h organoids. (M and N) Summary data of relative (M) aPKC-ζ III and (N) aPKC-ζ expression; Kruskal-Wallis test with Dunn’s multiple comparisons test, ^∗^*p* < 0.05, data are from *n* = 3 individual experiments. All graphs show mean ± SEM.

Our previous work assessed aPKC-ɩ and aPKC-ζ protein and mRNA expression in first-trimester and term human placentas and *in vitro* differentiated ST from primary isolated pCT (Shaha et al., 2022). However, single-cell RNA sequencing (scRNA-seq) and single-nuclei RNA sequencing (snRNA-seq) of early human placenta and human trophoblast organoids have revealed that multiple transcriptionally distinct pCT and ST states exist in the villous lineage (Arutyunyan et al., 2023; Keenen et al., 2025; Liu et al., 2018; Shannon et al., 2024; Vento-Tormo et al., 2018; Wang et al., 2024). These include a fusion-competent pCT population identified by the high expression of the trophoblast fusogens syncytin-1 and syncytin-2 (encoded by *ERVW-1* and *ERVFRD-1*, respectively), which are necessary for pCT fusion (Blaise et al., 2003; Frendo et al., 2003; Mi et al., 2000; Vargas et al., 2009). To understand if Par complex members are expressed in this critical state, we utilized the snRNA-seq data from first-trimester placentas previously presented by Wang et al. (2024). The dataset was visualized using uniform maniform approximation and projection (UMAP) dimensional reduction analysis. A total of 11 clusters were identified from 45,697 nuclei based on cell identification by analyzing marker gene expression for placental cell and trophoblast subtypes (Figure 1A; Figures S1A–S1E) (Duan et al., 2025; Keenen et al., 2025). Our analyses revealed four different cytotrophoblast (CT) states: bipotential pCTs (*BCAM*^+^, *ITGA6*^+^, and *GATA3*^*+*^), proliferative pCTs (*MKI67*^+^, *ITGA6*^+^, and *GATA3*^*+*^), pCTs (*ITGA6*^+^, *GATA3*^+^, and *MKI67*^−^), and fusion-competent pCTs (*ERVW-1*^+^, *ERVFRD-1*^*high*^, and *GREM2*^+^). We additionally identified two ST subtypes: early ST (*SDC1*^+^, *ERVW-1*^+^, and *ERVFRD-1*^low^) and ST (*PAPPA*^+^ and *SDC1*^+^).

Gene expression analyses revealed *PRKCI* expression in all pCT populations and ST, with the highest density of expression in pCTs, bipotential pCTs, and proliferative pCTs, confirming previous work by Bhattacharya et al., (2020) (Figure 1B). *PRKCZ* expression was observed in all pCT populations, with the highest density of expression in fusion-competent pCTs and ST (Figure 1C) (Shaha et al., 2022). *PARD3* was expressed in all pCT populations and ST, and like *PRKCZ*, the highest density of expression was observed in the fusion-competent pCTs (Figure 1D). *PARD6B* was expressed in all pCT populations, and in ST (Figure 1E). Thus, Par complex members are present in trophoblast villous lineage populations.

To understand if our previously published bioreactor-based trophoblast organoid model (Duan et al., 2025) recapitulated first-trimester pCT and ST populations, we performed snRNA-seq on organoids derived from human TSC CT27 and CT29 lines after 48 h of culture, a time point where a mixture of mononucleate pCT populations and multinucleate ST-like cells were present (Figure 1F). We identified four trophoblast clusters from 22,250 nuclei: proliferative CTs (*MKI67*^*+*^ and *ITGA2*^+^), pCTs (*GATA3*^+^ and *MKI67*^*-*^), ST (*ERVW-1*^+^, *ERVFRD-1*^low^, and *CGB3*^+*-*^), and a cluster that had features of both fusion-competent and early ST clusters in the analysis of first-trimester tissue (Figures 1G; Figures S2A–S2E). Unlike first-trimester tissue, no clusters showed a clear fusion-competent pCT state with high expression of *GREM2* and low expression of ST marker genes like *SDC1* and β-human chorionic gonadotropin-encoding genes (Figure S2). Rather, a large cluster was present with mixed expression of key fusion-competent marker genes, like *ERVFRD-1*, which is essential for trophoblast fusion (Vargas et al., 2009), and ST markers *SDC1* and *CGB2* (Figure S2). Together, these data suggest that the organoids recapitulate many signatures of pCT and ST nuclei in intact tissue.

Organoid snRNA-seq data were then used to examine the expression of Par complex components. Gene expression analyses for *PRKCI* revealed expression in pCT and ST populations (Figure 1H). *PRKCZ* was expressed in ∼70% of proliferative pCTs and pCTs at low levels, with ∼95% of fusion-competent/early ST cluster with higher expression than pCT sub-clusters, and ∼75% of the ST cluster displayed the highest level of expression observed (Figure 1I). *PARD3* and *PARD6B* were variably expressed in all clusters (Figures 1J and 1K). Interestingly, despite loss of *Prkcz* having no phenotypic impact on murine placental development, (Leitges et al., 2001) snRNA-seq expression profiles in both first-trimester tissue and organoids suggests that *PRKCZ*-encoded aPKC isoforms are strongly expressed in villous lineage CTs and are highly expressed in the critical fusion-competent state.

We previously observed a non-significant increase in aPKC-ζ III but not aPKC-ζ expression by western blotting and reverse-transcription PCR in first-trimester primary pCTs and *in vitro* differentiated ST (Shaha et al., 2022). To address if aPKC-ζ III is upregulated during pCT to ST differentiation in human trophoblast organoids, we assessed organoids after 24 and 48 h of rotational culture to capture a primarily mononucleate pCT population (24 h) and the progression toward ST-like cell states (48 h), and compared them to aPKC-ζ/-ζ III levels in undifferentiated TSCs (Figures 1F–1N). Like primary *in vitro* differentiated ST, we observed a consistent and significant 14-fold increase in the ∼55-kDa aPKC-ζ III band by western blotting, but not the 70-kDa aPKC-ζ band as cells progressed from TSCs to 48-h organoids (Figures 1L–1N). Therefore, *PRKCZ*-encoded isoforms increase in expression along the villous lineage and display high levels of expression in the critical fusion-competent pCT state, suggesting they may play a role in pCT to ST differentiation.

### aPKC-ζ III promotes trophoblast fusion

**Figure 2:**
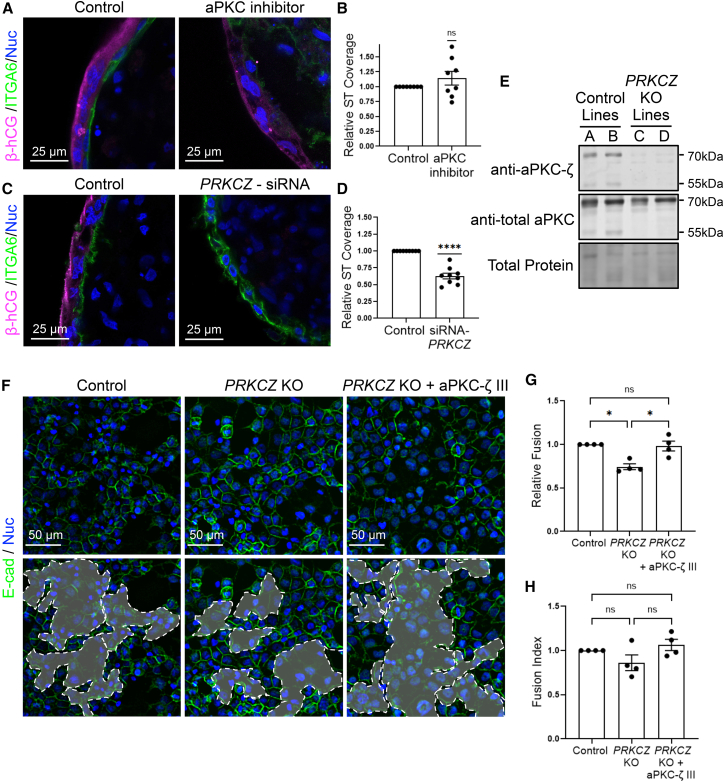
aPKC-ζ III regulates trophoblast fusion (A–D) Representative *xy*-plane images of 9- to 12-week placenta explants 48 h post ST -denudation of (A) control and aPKC inhibitor or (C) control and *PRKCZ*-siRNA-treated tissue stained for ITGA6 (green), β-hCG (magenta), and nuclei (blue), *n* = 8–9. Summary data of relative ST coverage of (B) aPKC inhibitor or (D) *PRKCZ* siRNA-treated explants; one-sample *t* test, ^∗∗∗∗^*p* ≤ 0.0001, data are from *n* = 8–9 placentas. (E) Representative western blot of control and *PRKCZ* KO BeWo cell lines with anti-aPKC-ζ and anti-total aPKC antibodies. (F) Representative images of E-cadherin (green) and nuclei (blue) in control and *PRKCZ* KO lines ± aPKC-ζ III rescue; dashed regions in lower panels indicate regions of multinucleated cells. (G) Summary data of relative fusion; Kruskal-Wallis test with Dunn’s multiple comparisons; ^∗^*p* ≤ 0.05, data are from *n* = 4 individual experiments. (H) Summary data for fusion index; Kruskal-Wallis test with Dunn’s multiple comparisons; data are from *n* = 4 individual experiments. All graphs show mean ± SEM.

To understand the contribution of aPKC isoforms during trophoblast fusion, we utilized multiple complementary *in vitro* models of pCT fusion and aPKC targeting strategies. We used an *ex vivo* first-trimester placental explant ST-intact model, a placental explant ST regeneration model that enriches a fusion-competent pCT subpopulation (Duan et al., 2025), primary first-trimester pCT, human TSC lines (Okae et al., 2018), BeWo trophoblastic cell lines that fuse and form ST-like multinucleate cells after treatment with cAMP analogs, and our trophoblast organoid model (Duan et al., 2025). Previous studies by Bhattacharya et al. identified that aPKC-ɩ promotes ST formation and that knockdown (KD) of aPKC-ɩ revealed a trending decrease in proliferation in the TSC stem state (Bhattacharya et al., 2020). Using an *ex vivo* floating first-trimester placental explant model with an intact ST, we treated with an aPKC inhibitor, which blocks the kinase activity of both aPKC-ι and aPKC-ζ, and has been shown to disrupt ST function (Mah et al., 2015; Riddell et al., 2018; Tsai et al., 2015; Patel et al., 2023). Aligning with previous studies, we found that inhibition of aPKC kinase activity decreased pCT proliferation (Figure S3) (Bhattacharya et al., 2020). We next used an *ex vivo* first-trimester placental explant ST regeneration model that enriches a fusion-competent pCT subpopulation to understand if aPKC inhibition decreased pCT fusion. First-trimester placental explants were denuded of ST, and exposed fusion-competent pCT was treated with aPKC inhibitor. Interestingly, when denuded explants were treated with aPKC inhibitor, there was no significant effect on ST regeneration (Figures 2A and 2B), suggesting that while pCT maintenance requires kinase activity, pCT fusion is independent of kinase activity. aPKC inhibitor treatment also had no effect on pCT fusion in primary isolated first-trimester trophoblasts induced to fuse for 72 h using 8-Br-cAMP in 2D culture (Figure S4).

To understand the contribution of *PRKCZ*-encoded aPKC-ζ isoforms during trophoblast fusion, first-trimester placental explants denuded of ST were treated with *PRKCZ*-targeting or control small interfering RNA (siRNA). *PRKCZ*-targeting siRNA treatment significantly reduced both aPKC-ζ and aPKC-ζ III expression in explant lysates (Figures S5A–S5C), and impaired ST regeneration (Figures 2C and 2D), suggesting the effects observed with *PRKCZ* KD are independent of kinase activity.

*PRKCZ* KD was also performed in CT29 TSC, and trophoblast organoids were subsequently formed. *PRKCZ*-siRNA treatment resulted in significant reductions in organoid aPKC-ζ and -ζ III (Figures S6A–S6C) expression, but pCT fusion was unchanged (Figures S6D and S6E). However, *PRKCZ* KD resulted in reduced expression of the ST marker gene *CGB*, trending decrease in *GCM1* expression, and a trending decrease in β-hCG secretion (Figures S6F–S6H), suggesting that in this model, disruption of *PRKCZ*-encoded proteins does not impact pCT fusion.

We used CRISPR-Cas9 technology to KO *PRKCZ* in the BeWo trophoblastic cell line (Figure S7) (Keryer et al., 1998; Wice et al., 1990). Western blotting revealed the loss of the 55-kDa band in two lines compared to two control lines using both an aPKC-ζ-specific and a total aPKC antibody, and loss of the 70-kDa band observed with the aPKC-ζ-specific antibody alone (Figure 2E). The total aPKC antibody detects both aPKC-ɩ and aPKC-ζ at 70 kDa; therefore, the 70-kDa band that persists in the *PRKCZ* KO lines represents the remaining aPKC-ɩ isoform. To determine if *PRKCZ* KO also leads to a decrease in fusion as observed in siRNA-treated explants, control and *PRKCZ* KO cells were induced to fuse. The proportion of multinucleate cells, but not fusion index, was significantly reduced in *PRKCZ* KO cells (Figures 2F–2H). The sum of the KD, KO, and aPKC inhibitor data across multiple models suggests that *PRKCZ*-encoded isoforms play a kinase activity-independent role in regulating pCT fusion.

aPKC-ζ III is predicted to have minimal kinase activity due to the absence of the PB1 domain; therefore, we hypothesized that it may be responsible for the altered pCT fusion we observed in our models. Plasmid-mediated reintroduction of aPKC-ζ III into the *PRKCZ* KO cells rescued fusion to control levels (Figures 2F and 2G), suggesting that aPKC-ζ III is the *PRKCZ* isoform regulating trophoblast fusion and identifying a function for this newly identified aPKC family member.

### Par-3 and aPKC-ζ III form stable interactions and promote trophoblast fusion

**Figure 3:**
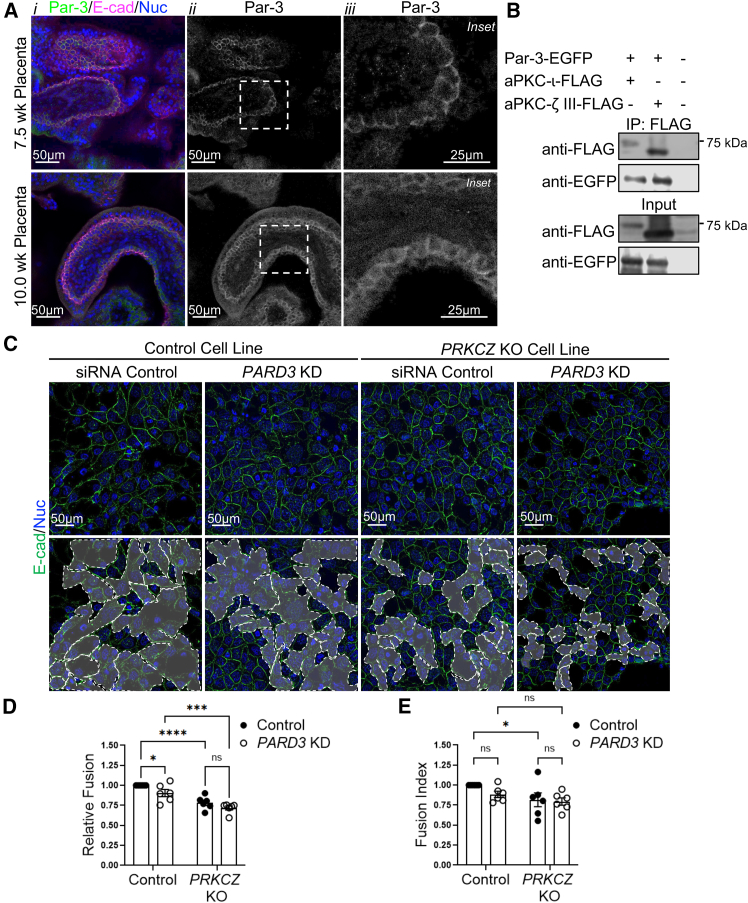
Par-3 interacts with aPKC-ζ III and promotes trophoblast fusion (A) Representative *xy*-plane images of first-trimester stained placenta tissue: (i) Par-3 (green), E-cad ([E-cadherin], magenta), and nuclei (blue); (ii) isolated Par-3 signal; and (iii) higher-magnification inset of isolated Par-3 signal; data are from *n* = 6 placentas. (B) Western blotting of FLAG immunoprecipitation of Par-3-EGFP and aPKC-ɩ-FLAG or aPKC-ζ III-FLAG; *n* = 4. (C) Representative images of E-cadherin (green) and nuclei (blue) in control and *PRKCZ* KO BeWo lines treated with control or *PARD3*-targetting siRNA; dashed regions in lower panels indicate regions of multinucleated cells. (D and E) Summary data of (D) relative fusion and (E) fusion index; two-way ANOVA with uncorrected Fisher’s least significant difference; mean ± SEM. ^∗^*p* ≤ 0.05, ^∗∗∗^*p* ≤ 0.001, ^∗∗∗∗^*p* ≤ 0.0001; data are from *n* = 6 individual experiments. All graphs show mean ± SEM.

To understand how aPKC-ζ III may be regulating pCT fusion, we examined if the canonical aPKC binding partner Par-3 interacts with aPKC-ζ III and influences pCT fusion, since aPKC-ζ III retains known Par-3-interacting domains (Shaha et al., 2022). Par-3 localization has not been reported in first-trimester placenta. Thus, to determine if Par-3 and aPKC-ζ isoforms localize to the same compartments, we examined Par-3 localization in mid to late first-trimester placental tissue. Par-3 signal was localized to pCT E-cadherin junctions and pCT cytoplasm, and inconsistent signal was also observed in ST and stromal cell populations (Figure 3A). The predominant localization of aPKC-ζ III is predicted to be cytoplasmic due to the lack of the PB1 domain required to interact with membrane-localized Par-6 (Dong et al., 2020). Transfection of aPKC-ζ III-EGFP into cells revealed that EGFP signal was restricted to the cytoplasm as predicted (Figure S8). We had previously reported a strong cytoplasmic anti-aPKC-ζ signal in villous trophoblasts (Shaha et al., 2022) and together, these data support that in first-trimester pCT, a substantial cytoplasmic pool of aPKC-ζ III and Par-3 exist. To determine if Par-3 and aPKC-ζ III interact, immunoprecipitations (IPs) were performed. Par-3-EGFP and aPKC-ɩ-FLAG (positive control) or aPKC-ζ III-FLAG were expressed in cells, and Par-3-EGFP was immunoprecipitated (Figure 3B). IPs revealed that aPKC-ζ III forms stable interactions with Par-3 (Figure 3B). As expected, canonical binding between Par-3 and aPKC-ɩ was also observed (Figure 3B). To determine if Par-3 is involved in trophoblast fusion in the same pathway as aPKC-ζ III, *PARD3* KD was performed in control and *PRKCZ* KO BeWo cells. Two different *PARD3*-targeting siRNAs significantly reduced Par-3 expression in BeWo (Figure S9). *PARD3* KD modestly reduced fusion in control lines, but not in *PRKCZ* KO lines (Figures 3C and 3D), although there was an additive effect of both *PARD3* KD and *PRKCZ* KO. This suggests Par-3 participates in multiple pathways that modulate trophoblast fusion and aPKC-ζ III acts downstream of Par-3. Together, our data confirm that Par-3 and aPKC-ζ III interact and are involved in trophoblast fusion.

### aPKC-ζ III modulates the Hippo signaling pathway to promote trophoblast fusion

Cytoplasmic Par-3 has been identified as a modulator of the Hippo signaling pathway by binding Hippo signaling components via its aPKC-binding domain (Lv et al., 2015). Specifically, Par-3 promotes the dephosphorylation of LATS1 (inactive) by recruiting protein phosphatase-1 (PP1A), resulting in dephosphorylation of YAP (active) and YAP nuclear translocation (Lv et al., 2015; Zhang et al., 2016). Hippo-YAP signaling has been established as a critical pathway for the maintenance of trophoblast stem state, and YAP1 is strongly expressed in both the nuclear and cytoplasmic compartments of pCT of first-trimester placenta (Meinhardt et al., 2020). In human TSC, YAP1-TEAD4 complexes promote trophoblast expansion by activating genes associated with trophoblast proliferation and also repress transcription of cell fusion and ST-promoting genes (Meinhardt et al., 2020; Shilei et al., 2022). Therefore, we assessed if *PRKCZ* isoforms are interacting with the Hippo signaling pathway. Although *PRKCZ* KD had no effect on fusion in our organoid model, bulk RNA sequencing results revealed that differential expression of *CCND1*, a proliferation-promoting cell-cycle gene involved in G1-S phase transition, increased in *PRKCZ* KD organoids (Figure S10A). *CCND1* is a direct downstream target gene of YAP1 in the Hippo signaling pathway (Figure S10A) (Mizuno et al., 2012; Shilei et al., 2022). The overall effect of *PRKCZ* KD in organoids was mild with only 16 significant differentially expressed genes observed, precluding Gene Ontology pathway and gene set enrichment analysis analyses (Figure S10A). Nuclear and cytoplasmic YAP1 signal was observed in control organoids at multiple time points during organoid maturation (Figure S10B). Interestingly, pseudotime analyses of trophoblast progenitor differentiation in the organoids using R package Monocle3 (Cao et al., 2019) predicted multiple pathways leading to ST nuclear states, including some trajectories that do not transit through the pre-fusion/early ST state, but transition directly from a pCT to an ST-like state (Figure S11). The imputation of similar differentiation trajectories that skip the pre-fusion pCT state has also been previously reported in scRNA-seq analyses of TSC Matrigel organoids (Shannon et al., 2024). Altogether, the combination of these data suggests that Hippo signaling is active in our trophoblast organoid model and may influence pCT differentiation and fusion, but *PRKCZ* isoforms do not influence fusion and are unlikely to modulate Hippo signaling in this model.

**Figure 4:**
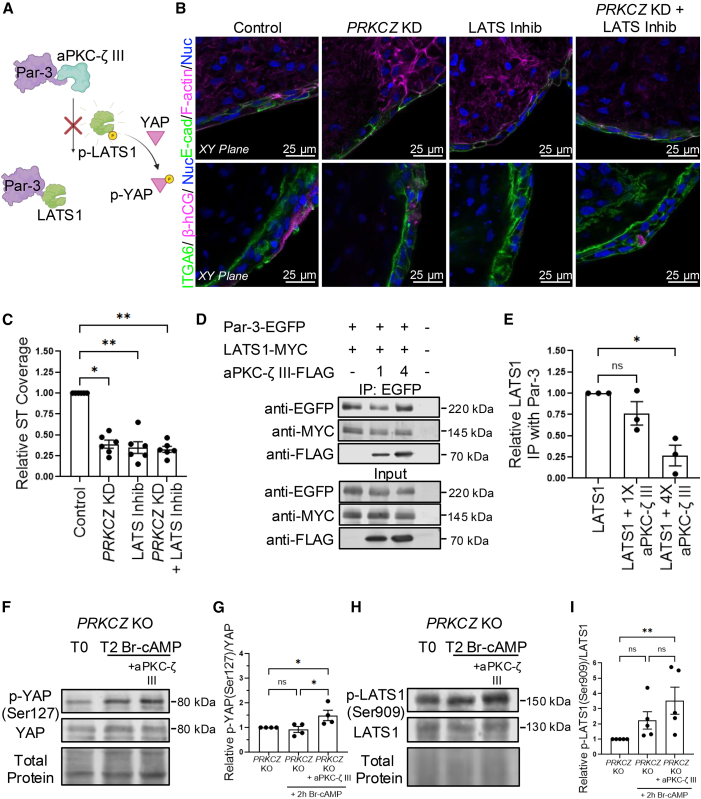
Hippo signaling is altered by aPKC-ζ III in trophoblasts (A) Graphical depiction of hypothesized interactions between aPKC-ζ III and Par-3 resulting in phosphorylation of YAP. (B) Representative *xy*-plane (top) and Z-projection (bottom) images of 9- to 12-week placenta explants 48 h post ST denudation and treated with *PRKCZ* siRNA, LATS inhibitor, or both stained for E-cad (E-cadherin; green), phalloidin (magenta), and nuclei (blue). (C) Summary data of relative ST coverage; Kruskal-Wallis test with Dunn’s multiple comparisons; ^∗^*p* ≤ 0.05, ^∗∗^*p* ≤ 0.01; data are from *n* = 6 placentas. (D) Western blotting of EGFP immunoprecipitation of Par-3-EGFP with LATS1-MYC ± aPKC-ζ III-FLAG. (E) Summary data of relative LATS1 immunoprecipitated with Par-3; Kruskal-Wallis test with Dunn’s multiple comparisons test; ^∗^*p* ≤ 0.05; data are from *n* = 3 individual experiments. (F and H) Representative western blot of (F) p-YAP (phospho-Ser127) and total YAP signal or (H) p-LATS (phospho-Ser909) and total LATS from *PRKCZ* KO cells at T = 0 (control) or T = 2 after Br-cAMP ± aPKC-ζ III reintroduction. (G and I) Summary data of relative (G) p-YAP/total YAP and (I) p-LATS1/total LATS1; Kruskal-Wallis test with Dunn’s multiple comparisons test; ^∗^*p* ≤ 0.05, ^∗∗^*p* ≤ 0.01; data are from *n* = 4–5 individual experiments. All graphs show mean ± SEM.

As previously mentioned, the aPKC-binding region of Par-3 is responsible for increasing the association of LATS1 and YAP1 with PP1A, resulting in decreased kinase activity of LATS1 and subsequent activation of YAP1 (Lv et al., 2015). Full-length aPKCs bind the Par-3 aPKC-binding region or PDZ2 domains via kinase and PBM domain interactions, both of which are conserved in aPKC-ζ III (Holly et al., 2020; Soriano et al., 2016; Thompson, 2022). Thus, we propose a model where aPKC-ζ III binding to Par-3 during pCT fusion and ST formation results in increased LATS1 activity and subsequent YAP phosphorylation and inactivation due to aPKC-ζ III outcompeting Hippo components for Par-3 binding (Figure 4A).

We utilized our first-trimester ST regeneration explant model to confirm that LATS inhibition (YAP1 activation) impaired fusion (Mizutani et al., 2022). We also sought to determine the effect of combined treatment of LATS inhibitor and *PRKCZ* KD on pCT fusion, because in this model *PRKCZ* KD had the strongest outcome on fusion. TDI-011536 is a derivative of the TRULI LATS inhibitor that blocks LATS kinase activity at nanomolar concentrations, thereby decreasing YAP1 phosphorylation and allowing for subsequent YAP1 nuclear localization (Kastan et al., 2022). Therefore, this inhibitor directly targets the regulatory point where aPKC-ζ III is proposed to influence the Hippo signaling pathway. ST-denuded placental explants were treated with *PRKCZ-*targeting siRNA, LATS inhibitor, or both for 48 h (Figures 4B and 4C). Control explants revealed the regeneration of multinucleated cells on the periphery of explants (Figure 4B). In *PRKCZ* KD explants and LATS inhibitor-treated explants, there was a 61% and 65% decrease in fusion, respectively, compared to controls, and no additive effect was observed with combined siRNA and inhibitor treatment (Figures 4B and 4C), suggesting aPKC-ζs and LATS are functioning at the same step of pCT fusion.

To directly test if aPKC-ζ III reduces LATS1 binding to Par-3, co-IPs were performed with exogenously expressed Par-3, LATS1, and aPKC-ζ III (Figure 4D). Addition of 1:1 ratio of LATS1-MYC:aPKC-ζ III-FLAG plasmid with Par-3-EGFP revealed a non-significant decrease in relative LATS1 immunoprecipitated with Par-3 (Figures 4D and 4E). When the ratio of LATS1-MYC:aPKC-ζ III-FLAG plasmid was increased to 1:4, there was a 73% decrease in the relative amount of LATS1 immunoprecipitated with Par-3 (Figures 4D and 4E), showing that aPKC-ζ III can outcompete LATS1 for Par-3 binding as hypothesized.

To confirm that aPKC-ζ III alters the Hippo signaling pathway during CT fusion, we reintroduced aPKC-ζ III into *PRKCZ* KO BeWo cells and examined phosphorylated levels of LATS1 and YAP. Phosphorylation of YAP at residue serine 127 promotes YAP cytoplasmic sequestration and inactivation (Zhao et al., 2007). aPKC-ζ III-FLAG was reintroduced into *PRKCZ* KO cells and then *PRKCZ* KO cells ± aPKC-ζ III-FLAG were treated with 8-Br-cAMP to induce fusion (Keryer et al., 1998). After 2 h, p-Ser127-YAP remained unchanged in *PRKCZ* KO cells treated with 8-Br-cAMP but increased 1.5-fold in *PRKCZ* KO cells when aPKC-ζ III was rescued relative to untreated controls (Figures 4F and 4G). Similar experiments were performed to analyze LATS1 phosphorylation at Ser909, essential for LATS1 activation and kinase activity (Chan et al., 2005). Treatment with 8-Br-cAMP alone resulted in a non-significant increase of p-LATS1(Ser909) after 2 h, which increased 3-fold when aPKC-ζ III was reintroduced (Figures 4H and 4I). These results indicate that aPKC-ζ III modulates the Hippo signaling pathway by reducing LATS1 binding to Par-3 and subsequently inactivates YAP1 to promote pCT fusion and ST differentiation in trophoblasts.

## Discussion

The first trimester of pregnancy is a critical period of development for the human placenta involving the establishment and maintenance of pCT and ST populations. ST malformation underlies many pregnancy complications; thus, understanding the molecular mechanisms that contribute to pCT to ST differentiation remains crucial to identifying therapies. Here, snRNA-seq analyses of human placenta and human trophoblast organoids identify that *PRKCZ* expression is highest in the villous lineage, with elevated expression in fusion-competent pCTs/early ST and ST. These data highlight that *PRKCZ* isoforms likely play a role in ST formation, specifically at the fusion competency stage. Using multiple models of pCT fusion, we identified that *PRKCZ* isoforms play an important role in this critical process. Fusion in *PRKCZ* KO cells was rescued by the reintroduction of aPKC-ζ III, revealing that aPKC-ζ III is the aPKC-ζ isoform necessary for regulation of pCT fusion. Finally, we identified that aPKC-ζ III reduces LATS1 binding to Par-3, thereby promoting LATS1 activity, inactivating YAP1, and establishing a pathway via which aPKC-ζ III regulates ST formation. Our work is the first to establish a key regulator of Hippo signaling in trophoblasts, and we have discovered that this previously unidentified aPKC isoform promotes pCT fusion by activating the Hippo signaling pathway to control cell fate.

The expansion of models to study ST differentiation and advancement of RNA sequencing technologies has led to the identification of multiple distinct pCT states during TSC to ST differentiation (Haider et al., 2018; Keenen et al., 2025; Liu et al., 2018; Okae et al., 2018; Turco et al., 2018; Vento-Tormo et al., 2018; Wang et al., 2024). scRNA-seq has been performed on human placental tissue and organoid models; however, due to the need for single-cell dissociation, the most differentiated villous trophoblast populations captured are mononucleate precursor ST with very few multinucleate ST (Hua et al., 2024; Liu et al., 2018; Shannon et al., 2024; Vento-Tormo et al., 2018; Zhuang et al., 2023). snRNA-seq has only recently been performed in human placental tissue, capturing for the first time the genetic heterogeneity within the multinucleated ST (Keenen et al., 2025; Wang et al., 2024). The availability of multiple different models has unveiled the potential to recapitulate different pCT states, each with their own strengths to model transitionary stages during ST differentiation (Keenen et al., 2025; Shannon et al., 2024; Sheridan et al., 2021). Our data highlight how molecules may contribute to ST differentiation at specific steps, and that using a combination of models is critical to address the role of individual molecules in villous differentiation. Previous studies identified that aPKC-ɩ is important for human TSC to ST differentiation, and we observed the highest density of *PRKCI* expression in bipotential cells in our snRNA-seq analysis of first-trimester tissue, whereas *PRKCZ* isoforms and *PARD3* mRNA are upregulated in fusion-competent pCTs (Figures 1A–1C) (Bhattacharya et al., 2020; Wang et al., 2024). This suggests that aPKC-ι may play a regulatory role earlier in the differentiation pathway toward ST, whereas aPKC-ζ isoforms and Par-3 modulate the critical fusion competence regulatory point during differentiation (Figures 1B and 1C). Therefore, with the availability of numerous models in the trophoblast field, it will be important for researchers to use a combination of methods to interrogate the full spectra of regulatory pathways that may occur *in vivo* and contribute to trophoblast differentiation.

snRNA-seq of human first-trimester placenta tissue from Wang et al. and bioreactor trophoblast organoids revealed that while organoids remain an excellent 3D model to understand complex interactions between cells, not all pCT subtypes are represented in high abundance in this model (Wang et al., 2024). This may have contributed to our inability to observe a transcriptionally distinct cluster of fusion-competent pCT in bioreactor organoids, or this could be due to rapid transit through the fusion-competent pCT state. Time-dependent sequencing of trophoblast organoids could yet reveal a coherent fusion-competent cluster. Critically, consistent with other TSC-based organoid models, our pseudotime analyses suggest that CT27 and CT29 TSC organoid models may bypass a pre-fusion/early ST state (Figure S11) (Shannon et al., 2024). Using scRNA-seq data to model villous lineage trajectory, Shannon et al. observed that when TSC lines are used to produce Matrigel-based trophoblast organoids, TSCs may bypass pCT states and differentiate directly into ST (Shannon et al., 2024). Interestingly, their data also revealed that primary cell-derived Matrigel trophoblast organoids more faithfully represented *in vivo* villous lineage trajectory modeling. Therefore, data are consistently suggesting that the widely available TSC lines may use additional signaling pathways beyond those predicted using tissue and primary cells to form ST. This is congruent with our data showing that *PRKCZ* KD did not impair ST fusion in the organoids despite reproducible effects on ST formation in explant cultures that rely on primary pCT. The difference in our results between models can be because a spectrum of pathways regulates this key process and in some contexts, the role of aPKC-ζ III in regulating fusion can be circumvented. But alternatively, we cannot rule out that the residual expression of aPKC-ζ and/or aPKC-ζ III after KD was able to maintain the level of signaling required for trophoblast fusion in the organoid model. However, why this is not the case in the explant model, where we also had residual protein expression after KD, leaves the possibility open that there are multiple context-dependent regulators of LATS1 activity that can ultimately influence fusion.

Interestingly, while inhibiting aPKC kinase activity reduced explant pCT proliferation, it did not impair explant and primary first-trimester pCT fusion, suggesting that aPKCs have a complex regulation that is contextually dependent on whether pCTs are maintaining their stem state or undergoing fusion. While it remains unknown how aPKCs regulate the pCT stem state, they are known to influence stem cell differentiation through multiple mechanisms and have been shown to modulate Hippo signaling in a stepwise, context-dependent manner (Archibald et al., 2015; Hirate et al., 2015)^.^ Thus, understanding how aPKCs promote the pCT stem state and if they regulate Hippo signaling will be important for future studies.

*PRKCZ* KD, but not inhibition of aPKC kinase activity, reduced pCT fusion, revealing a kinase-independent role of aPKCs in the human placenta. aPKC-ζ III likely only has basal levels of activity due to its inability to become activated via Par-6-mediated interactions. Graybill et al. created an aPKC mutant without a PB1 domain (only 2 amino acids smaller than aPKC-ζ III) and revealed the mutant lacked kinase activity, supporting our hypothesis by suggesting that aPKC-ζ ΙΙΙ can participate in cell signaling by modulating binding interactions without the ability to phosphorylate targets (Graybill et al., 2012). Importantly, the aPKC inhibitor used in this study blocks both aPKC-ζ and aPKC-ι activity, suggesting that both of these isoforms do not impact pCT fusion, or that they have opposing actions at this regulatory point (Patel et al., 2023; Riddell et al., 2018). Since reintroduction of aPKC-ζ III alone in *PRKCZ* KO cells rescued fusion, it seems likely that the activity of both aPKC-ι and -ζ are not necessary at this point in ST formation, but further experiments are necessary to fully address the role of all aPKC isoforms and kinase-independent binding contributions. Our IP data revealed that aPKC-ζ III must be in excess abundance to LATS1 to significantly impair LATS1 interaction with Par-3. In the mouse brain, PKM-ζ (a brain-specific *PRKCZ*-encoded aPKC-ζ isoform) competes with aPKC-λ (aPKC-ɩ homolog) for binding to Par-3 to suppress axon specification; however, selective silencing of PKM-ζ allows for the maturation of a single axon (Parker et al., 2013). We suspect the upregulation of aPKC-ζ III is a critical step during villous lineage differentiation that is required to allow aPKC-ζ III to outcompete other cytoplasmic Par-3-binding partners to allow for Hippo signaling to occur.

snRNA-seq data revealed an apparent stepwise increase in expression of *PRKCZ* mRNA from bipotential pCTs, pCTs, to fusion-competent pCTs, suggesting there is an unknown regulatory stimulus controlling aPKC-ζ III expression. A stepwise increase in the expression of aPKC-ζ III was also seen in our TSC organoid model. Currently, it is not known if aPKC-ζ III-encoding mRNA is transcribed from an alternative promoter sequence or via alternative splicing alone. In neurons, the expression of aPKC-ζ and PKM-ζ is epigenetically regulated via histone acetylation and DNA methylation (Borodinova et al., 2019; Pramio et al., 2023). PKM-ζ is transcribed from an internal promoter sequence with a CREB-binding site that is demethylated in differentiated neurons (Pramio et al., 2023). Interestingly, CREB has been shown to play an important role in pCT differentiation, directly regulating *GCM1*, a key transcriptional regulator of ST formation (Chang et al., 2005; Jeyarajah et al., 2022; Schubert et al., 2008). Therefore, understanding if aPKC-ζ ΙΙΙ is regulated via this alternative promoter and, therefore, sensitive to cAMP/CREB activation will be important to examine in the future. The observed increased and sustained expression of *PRKCZ* mRNA and aPKC-ζ III protein in ST also highlights that this form of aPKC may have other critical roles in ST. We previously observed that antibodies raised against aPKC-ζ isoforms reveal a strong cytoplasmic ST signal and a cytoplasmic signal within first-trimester pCT (Shaha et al., 2022). Together with the exclusive localization of aPKC-ζ III-GFP to the cytoplasm observed here, we can infer that aPKC-ζ III localizes to the cytoplasm in pCT and the ST (Shaha et al., 2022). Others have determined that cytoplasmic localization of aPKC is usually found during interphase and that the membrane targeting of aPKC is required for mitosis and progression of the cell cycle (Jones et al., 2023)^.^ Disruption of aPKC binding to Par-6 by deletion of the PB1 domain leads to a cytoplasmic localization in neuroblasts (Jones et al., 2023). While the function of cytoplasmic aPKC was not examined, since the ST does not undergo mitosis, it is possible that aPKC-ζ III plays a similar role in this cell type. Interestingly, other groups have identified that the hinge domain of aPKC-ɩ and aPKC-ζ specifically recruits the domain to the nucleus or cytoplasm, respectively, revealing the aPKC isoforms have inherent subcellular localization signals (Seidl et al., 2012). Without a polarized distribution of aPKC-ζ III, it is unlikely to directly regulate cell polarity in the ST, although indirect regulation of polarity by competing for Par-3 binding may be possible.

Importantly, our work has shown a potentially trophoblast-specific regulatory point for the Hippo signaling pathway. Hippo signaling is a ubiquitous pathway, and dysregulation has been well associated with numerous diseases such as immune dysfunction, cardiac disease, and cancer (Dey et al., 2020; Fu et al., 2022; Han, 2019). aPKCs have also been recognized as critical regulators of tumorigenesis and can have cell- and cancer-dependent tumor-promoting or -suppressive roles (Reina-Campos et al., 2019). Increased expression of aPKC-ζ has been found to promote breast cancer (Paul et al., 2015), colorectal cancer (Islam et al., 2018), and pancreatic cancer (Butler et al., 2013). Additionally, *PRKCZ* splice variants have been identified in prostate cancer (Yao et al., 2012). Reactivation of placental-specific genes and pathways has become increasingly observed in cancer (Novakovic and Saffery, 2013; Rousseaux et al., 2013). Like the placenta, cancer cells have the ability to become invasive via epithelial-mesenchymal transition, induce tolerance of the immune system, and become multinucleated via the reactivation of genes encoding the syncytins, the retrovirally co-opted trophoblast fusogens (Bjerregaard et al., 2006; Novakovic and Saffery, 2013; Rousseaux et al., 2013). The potential for reactivation of aPKC-ζ III expression in pathology and the role it could play in modulating Hippo signaling in other tissues and cell types is another interesting future direction from this work.

Here, we have identified, for the first time, a placental-specific activator of Hippo signaling. Dysregulation of Hippo signaling has been established in trophoblast dysfunction, resulting in placental pathologies (Home et al., 2012; Lin et al., 2023; Liu et al., 2020; Soncin and Parast, 2020; Wu et al., 2024). Preeclampsia and intrauterine growth restriction are serious pregnancy disorders that complicate 2%–8% of all pregnancies. While the etiology remains unclear, these complications are thought to originate from the placenta (Huppertz, 2008). Trophoblast fusion and expression of fusion competency machinery are impaired in both preeclampsia and intrauterine growth restriction; thus, understanding the molecular regulators of trophoblast fusion is critical (Chen et al., 2006; Langbein et al., 2008; Ruebner et al., 2010). Our work highlights the importance of discovering fundamental human biology and placental-specific pathways for the development of therapies to treat placental pathologies. Thus, future studies determining how aPKC-ζ III expression is regulated and what additional roles it plays in villous trophoblasts may have widespread implications for human development and pathogenesis.

## Resource availability

### Lead contact

The lead contact for this study is Meghan Riddell. All requests for reagents and methods should be directed to and will be fulfilled by the lead contact (mriddell@ualberta.ca).

### Materials availability

Plasmids generated from this study can be purchased from Vectorbuilder. *PRKCZ* KO lines can be requested from lead contact.

### Data and code availability

Bulk RNA-seq and snRNA-seq data generated from this study have been deposited in the Gene Expression Omnibus with the accession GSE310653. There are no restrictions on data availability use. snRNA-seq data can be found at https://riddell-lab.shinyapps.io/single_nuclei_placenta/.

## Acknowledgments

We would like to thank the patients who donated tissue to our study and the staff of the Woman’s Health Options Clinic for the help in accessing this critical resource. We would also like to thank Mike Wong in the Advanced Cell Exploration Core for support with the bulk RNA sequencing and CRISPR-Cas 9 experiments. M.R. is supported by the Canada Research Chairs program, and support for equipment was provided to M.R. by the Canada Foundation for Innovation. S.Z.S received salary support from Alberta Innovates and Advanced Education and Women and Children’s Health Research Institute Graduate studentships. W.K.D was supported by the Natural Sciences and Engineering Research Council of Canada and Women and Children's Health Research Institute studentships. Bulk RNA-seq library preparation was performed by the University of Alberta Faculty of Medicine & Dentistry High Content Analysis Core (RRID:SCR_019182). Flow Cytometry Facility experiments were performed at the University of Alberta Faculty of Medicine & Dentistry Flow Cytometry Facility, RRID:SCR_019195. Cell Imaging Core experiments were performed at the University of Alberta Faculty of Medicine & Dentistry Cell Imaging Core, RRID:SCR_019200. Plasmid sequencing was performed at the University of Alberta Faculty of Medicine & Dentistry Advanced Cell Exploration Core, RRID:SCR_019182. Advanced Cell Exploration Core Experiments were performed at the University of Alberta Faculty of Medicine & Dentistry Advanced Cell Exploration Core, RRID:SCR_019182. Cell Imaging Core Experiments were performed at the University of Alberta Faculty of Medicine & Dentistry Cell Imaging Core, RRID:SCR_019200. This work was supported through the Natural Sciences and Engineering Research Council of Canada Discovery Grants Program (RGPIN-2021-02807), Women and Children’s Health Research Institute and their donors the Alberta Women’s Health Foundation and the Stollery Children’s Hospital Foundation (2863), the Canada Research Chairs Program (CRC-2023-00055), and a One Child Every Child project supported by the Canada First Research Excellence Fund.

## Author contributions

S.Z.S., W.K.D., and M.R. conceptualized the project. M.R. obtained funding for the project. S.Z.S., W.K.D., and I.K.D. performed experiments and analyzed data. J.G.R. performed all bioinformatic analyses. S.Z.S. and M.R. wrote the original manuscript with comments and edits from all authors. M.R. performed supervision.

## Declaration of interests

The authors declare no competing interests.

## STAR★Methods

### Key resources table

**Table:** 

REAGENT or RESOURCE	SOURCE	IDENTIFIER
**Antibodies**		

Anti-CD49f (clone GoH3) Rat mAb [IF: 1:200]	Stemcell Technologies	Cat #60037; Lot: SC10713. RRID: AB_3717553
Anti-E-cadherin (clone 180215) Mouse mAb [IF: 1:400]	R&D systems	Cat# MAB18381; Lot: JAT022008, JAT0221051, JAT0222121; RRID: AB_2076805
Anti-hCG beta (clone 5H4-E2) Mouse mAb [IF: 1:200]	Abcam	Cat# ab9582; Lot: GR3300377-1, GR3425916-1; RRID: AB_296507
Anti- Ki-67 (8D5) Mouse mAb [IF 1:200]	Cell Signaling Technology	Cat#9449; Lot: 12; RRID: AB_2797703
Anti-PARD3 Rabbit pAb [IF 1:200, WB: 1:2000]	Atlas Antibodies	Cat# HPA030443; Lot: 13543; RRID: AB_10600926
Anti-PKC zeta (H-1) Mouse mAb [WB: 1:10,000] Targets total aPKC	Santa Cruz Biotechnology	Cat# sc-17781; Lot: C1122; RRID: AB_628148
Anti-PRKCZ Rabbit pAb [WB: 1:8000, IF: 1:200]	Sigma-Aldrich	Cat# HPA021851; Lot: A118749; RRID: AB_1855433
Anti-DYKDDDDK Tag (D6W5B) Rabbit mAb [WB:	Cell Signaling Technology	Cat# 70569; Lot: 7; RRID: AB_2799005
Anti-GFP (Green Fluorescent Protein) Rabbit pAb [WB: 1.75μg/mL]	MBL International	Cat# 598; Lot: 84; RRID: AB_591819
Anti-Phospho-LATS1 (Ser909) Rabbit mAb [WB: 1:2000]	Cell Signaling Technology	Cat# 9157; Lot:2; RRID: AB_2133515
Anti- LATS1 (clone C66B5) Rabbit mAb [WB: 1:2000]	Cell Signaling Technology	Cat# 3577; Lot:9; RRID: AB_2133513
Anti-Myc-Tag (clone 9B11) Mouse mAb [WB 1:1000]	Cell Signaling Technology	Cat# 2267; Lot: 24; RRID: AB_331783
Anti- Phospho-YAP (Ser127) Rabbit pAb [WB: 1:2000]	Cell Signaling Technology	Cat# 4911; Lot: 5; RRID: AB_2218913
Anti- YAP (clone D8H1X) XP Rabbit mAb [WB: 1:2000; IF: 1:200]	Cell Signaling Technology	Cat# 14074; Lot: 5; RRID: AB_2650491)
Donkey anti-Mouse IgG Secondary Antibody, Alexa Fluor™ 488 [IF: 1:400]	Invitrogen	Cat# A-21202; RRID:AB_141607
Donkey anti-Rabbit IgG Secondary Antibody, Alexa Fluor™ 488 [IF: 1:400]	Invitrogen	Cat# A-21206; RRID:AB_2535792
Donkey anti-Mouse IgG Secondary Antibody, Alexa Fluor™ 594 [IF: 1:400]	Invitrogen	Cat# A-21203; RRID:AB_2535789
Donkey anti-Rabbit IgG Secondary Antibody, Alexa Fluor™ 594 [IF: 1:400]	Invitrogen	Cat# A-21207; RRID:AB_141637
Goat anti-Rat IgG Secondary Antibody, Alexa Fluor™ 488 [IF: 1:400]	Invitrogen	Cat# A-11006; RRID:AB_2534074
Goat anti-Rabbit IgG Secondary Antibody, Alexa Fluor™ 750 [WB: 1:10,000]	Invitrogen	Cat# A-21039; RRID:AB_2535710
Goat anti-Mouse IgG Secondary Antibody, Alexa Fluor™ 680 [WB: 1:10,000]	Invitrogen	Cat# A-21057; RRID:AB_2535723
**Biological samples**		

Human Placenta 10 Weeks	This Study	Sample 1
Human Placenta 10 Weeks	This Study	Sample 2
Human Placenta 11.5 Weeks	This Study	Sample 3
Human Placenta 10.5 Weeks	This Study	Sample 4
Human Placenta 9.3 Weeks	This Study	Sample 5
Human Placenta 10 Weeks	This Study	Sample 6
Human Placenta 9 Weeks	This Study	Sample 7
Human Placenta 11 Weeks	This Study	Sample 8
Human Placenta 11 Weeks	This Study	Sample 9
Human Placenta 10 Weeks	This Study	Sample 10
Human Placenta 11 Weeks	This Study	Sample 11
Human Placenta 11 Weeks	This Study	Sample 12
Human Placenta 10.5 Weeks	This Study	Sample 13
Human Placenta 11 Weeks	This Study	Sample 14
Human Placenta 10 Weeks	This Study	Sample 15
Human Placenta 10.3 Weeks	This Study	Sample 16
Human Placenta 10 Weeks	This Study	Sample 17
Human Placenta 12.7 Weeks	This Study	Sample 18
Human Placenta 10.5 Weeks	This Study	Sample 19
Human Placenta 10.5 Weeks	This Study	Sample 20
Human Placenta 11 Weeks	This Study	Sample 21
Human Placenta 12.3 Weeks	This Study	Sample 22
Human Placenta 9 Weeks	This Study	Sample 23
Human Placenta 9 Weeks	This Study	Sample 24
Human Placenta 10 Weeks	This Study	Sample 25
Human Placenta 5 Weeks	This Study	Sample 26
Human Placenta 8 Weeks	This Study	Sample 27
Human Placenta 10 Weeks	This Study	Sample 28
Human Placenta 9 Weeks	This Study	Sample 29
Human Placenta 7.5 Weeks	This Study	Sample 30
Human Placenta 8.5 Weeks	This Study	Sample 31
Human Placenta 5.5 Weeks	This Study	Sample 32
Human Placenta 6 Weeks	This Study	Sample 33
Human Placenta 7 Weeks	This Study	Sample 34
Human Placenta 7.5 Weeks	This Study	Sample 35
Human Placenta 6 Weeks	This Study	Sample 36
Human Placenta 6.5 Weeks	This Study	Sample 37
Human Placenta 7.5 Weeks	This Study	Sample 38
Human Placenta 6 Weeks	This Study	Sample 39
Human Placenta 6 Weeks	This Study	Sample 40
Human Placenta 6 Weeks	This Study	Sample 41
Human Placenta 6 Weeks	This Study	Sample 42
Human Placenta 6 Weeks	This Study	Sample 43
Human Placenta 6.5 Weeks	This Study	Sample 44

**Chemicals, peptides, and recombinant proteins**		

Advanced DMEM/F12	Gibco	Cat# 12634-101
DMEM/F12	Gibco	Cat# 11320033
2-Mercaptoethanol	Sigma-Aldrich	Cat# M3148
Fetal Bovine Serum	Wisent, Multicell Inc.	Cat# 098150; Lot: 185730
Primocin	InvivoGen	Cat# ant-pm-05
Bovine Serum Albumin	Sigma-Aldrich	Cat#A9085
Insulin-Transferrin-Selenium-Ethanolamine (ITS -X) (100×)	Gibco	Cat# 51500056
L-Ascorbic acid	Sigma-Aldrich	Cat# A5960; CAS: 50-81-7
Human EGF	Peprotech	Cat# AF-100-15
CHIR 99021	BioGems	Cat# 2520691; CAS: 252917-06-9
A 83-01	BioGems	Cat# 9094360; CAS: 909910-43-6
SB 431542 hydrate	Sigma-Aldrich	Cat# 616464; CAS: 301836-41-9
Y-27632 Dihydrochloride	BioGems	Cat# 1293823; CAS: 129830-38-2
Valproic acid sodium salt	BioGems	Cat# 1066656; CAS: 1069-66-5
*N*-2 Supplement	Gibco	Cat# 17502048
B-27 supplement, minus vitamin A	Gibco	Cat# 12587010
L-Glutamine	Gibco	Cat# 21051-024; CAS: 56-85-9
Human R-Spondin-1	Peprotech	Cat# 120-38
Prostaglandin E2	BioGems	Cat# 3632464; CAS: 363-24-6
Human HGF Protein	Peprotech	Cat# 100-39
Human FGF-basic	Peprotech	Cat# 100-18C
N-acetyl-L-cysteine	Sigma-Aldrich	Cat# A9165; CAS: 616-91-1
Collagen IV, Mouse	Corning	Cat# CB-40233
Iscove’s Modified Dulbecco’s Medium	Gibco	Cat# 12440061
Ham’s F-12 Nutrient Mix	Gibco	Cat# 11765047
Gentamycin	Gibco	Cat# 15750-060
Penicillin-Streptomycin	Gibco	Cat# 15140122
8-Bromoadenosine 3′,5′-cyclic monophosphate sodium salt	Sigma-Aldrich	Cat# B7880; CAS: 76939-46-3
Lipofectamine LTX reagent with PLUS reagent	Invitrogen	Cat# 15338100
0.25% trypsin-EDTA	Gibco	Cat# 25200-056
TrypLE™ Express Enzyme	Gibco	Cat# 12604013
Opti-MEM™ I Reduced Serum Medium	Gibco	Cat# 31985062
Nuclease-Free Duplex Buffer	IDT	Cat# 11-01-03-01
Lipofectamine CRISPRMAX Transfection Reagent	Invitrogen	Cat# CMAX00001; Lot: 2634820
Cas9 Plus Reagent	Invitrogen	Cat# 100035624; Lot: 2634802
Lipofectamine CRISPRMAX Reagent	Invitrogen	Cat# 100035629; Lot: 2650857
Alt-R S.p. Cas9 nuclease V3	IDT	Cat#1081058, Lot: 0000833182
myristoylated aPKC pseudosubstrate inhibitor	Invitrogen	Cat# 77749
TDI-011536; LATS inhibitor	MedChemExpress	Cat# HY-150042; CAS: 2687970-96-1
Protease Inhibitor Cocktail	Sigma-Aldrich	Cat# P2714
Halt Phosphatase Inhibitor Cocktail	Thermoscientific	Cat# 78420
Bovine Gelatin	Sigma-Aldrich	Cat# G1393
Sigmacote	Sigma-Aldrich	Cat# SL2
Normal Donkey Serum	Sigma-Aldrich	Cat# S30
Human IgG Isotype Control	Invitrogen	Cat# 02-7102
Fluoromount-G	SouthernBiotech	Cat# 0100-01
TRIzol Reagent	Invitrogen	Cat# 15596026
SYBR Green Universal Master Mix	Applied Biosystems	Cat# 4309155
RedSafe Nucleic Acid Staining Solution	FroggaBio	Cat# 21141

Phalloidin iFluor 594	AAT Bioquest	Cat# 23122
**Critical commercial assays**		

Chromium Nuclei Isolation Kit with RNase Inhibitor	Novogene	Cat# 1000494
Pierce BCA Assay Kit	Thermo Fisher Scientific	Cat# 23227
β-hCG ELISA kit	DRG International	Cat# EIA-1911
PureLink RNA Mini Kit	Invitrogen	Cat# 12183025
iScript cDNA Synthesis Kit	Bio-Rad	Cat# 1708890
PCR SuperMix	Invitrogen	Cat# 10572014

**Deposited data**		

First trimester placenta snRNA-seq data	(Wang et al., 2024)	GEO: GSE247038
TSC organoid bulkRNA-seq data	This Study	GEO: GSE310653
TSC organoid snRNA-seq data	This Study	GEO: GSE310653

**Experimental models: Cell lines**		

Human trophoblast stem cell (CT27)	Riken Cell Bank	RCB4936; RRID:CVCL_A7AZ
Human trophoblast stem cell (CT29)	Riken Cell Bank	RCB4937; RRID:CVCL_A7BA
BeWo		RRID:CVCL_0044
HEK293T		RRID:CVCL_0063

**Oligonucleotides**		

ON-TARGETplus Human *PRKCZ* (5590) siRNA	Dharmacon	Cat# J-003526-14
ON-TARGETplus Human *PARD3* (56288) siRNA	Dharmacon	Cat# J-015602-06
non-targeting control siRNA	Dharmacon	Cat# D-001810-10
CRISPR Guide RNAs: 5′ tccagta gacgacaaga a 3′	IDT	N/A
Alt-2 Cas9 Neg Ctrl crRNA #1	IDT	Cat# 1072544; Lot#0000828510
Alt-R CRISPR Cas9 tracrRNA-ATTO 550	IDT	Cat# 1075927; Lot: 00005558022
RT-PCR Primer for *CGB* (5’->3′) F: GCCTCATCCTTGGCGCTAGA R: TATACCTCGGGGTTGTGGGG	IDT	N/A
RT-PCR Primer for *GCM1* (5’->3′) F: GTGCTGTCTGCTTCTCCGTA R: GATAAGGTCAGGCCAGCCAA	IDT	N/A
RT-PCR Primer for *SRY* (5’->3′) F: CAGATCCCGCTTCGGTACTC R: TTTGTCCAGTGGCTGTAGCG	IDT	N/A
RT-PCR Primer for *RNA18SN1* (5’->3′) F: GCAATTATTCCCCATGAACG R: GGCCTCACTAAACCATCCAA	IDT	N/A

**Recombinant DNA**		

aPKC-ζ III-FLAG plasmid	Vector Builder	Vector Builder ID: VB230324-1471jgr
aPKC-ζ III-EGFP plasmid	Vector Builder	Vector Builder ID: VB230324-1483nyd
pEGFP-N1-Par3 plasmid	(Hikita et al., 2018)	Dr. Masanori Nakayama
pcDNA3 Lats1 (Nigg HS189) (LATS1-Myc Tag)	Addgene	Plasmid #: 41156; RRID:Addgene_41156

**Software and algorithms**		

Volocity Imaging Software V 7.0.0	Quorum Technologues	https://www.volocity4d.com/bio
cellSens Dimensions Imaging Software V 1.11	Olympus	www.olympus-sis.com
ImageJ	NIH	https://imagej.net/ij/
ImageStudio V 6.0.0.28	LicorBio	https://www.licorbio.com/image-studio
QuantStudio Design & Analysis Software V 1.5.1	Thermo Fisher Scientific	https://www.thermofisher.com/ca/en/home/technical-resources/software-downloads/quantstudio-3-5-real-time-pcr-systems.html
GraphPad PRISM V 10.4.2	GraphPad	https://www.graphpad.com/features
BioTek Gen 5 Software for Detection	Agilent Technologies	https://www.agilent.com/en/product/microplate-instrumentation/microplate-instrumentation-control-analysis-software/imager-reader-control-analysis-software/biotek-gen5-software-for-detection-1623227?srsltid=AfmBOoqJWOFPtrhNw1DBhBhwslgPx8v-ZxFoDMJqMJhbHzXmG6h1mCnu
RStudio version 2025.09.1 + 401	Posit	https://posit.co/download/rstudio-desktop/
R version 4.5.1	R Project	https://www.r-project.org/
Ubuntu 22.04.3	Ubuntu	https://ubuntu.com/
FastQC 0.11.9	Babraham Bioinformatics	https://www.bioinformatics.babraham.ac.uk/projects/fastqc/
STAR 2.7.11	(Dobin et al., 2013)	https://github.com/alexdobin/STAR
Rsubread 2.22.1	(Liao et al., 2019)	https://bioconductor.org/packages/release/bioc/html/Rsubread.html
DESeq2 1.48.2	(Love et al., 2014)	https://bioconductor.org/packages/release/bioc/html/DESeq2.html
clusterProfiler 4.16.0	(Wu et al., 2021; Xu et al., 2024; Yu, 2024; Yu et al., 2012)	https://bioconductor.org/packages/release/bioc/html/clusterProfiler.html
CellRanger 9.0.1	(Zheng et al., 2017)	https://www.10xgenomics.com/support/software/cell-ranger/latest
Seurat 5.3.0	(Hao et al., 2024)	https://satijalab.org/seurat/
Monocle3 1.4.26	(Trapnell et al., 2014)	https://cole-trapnell-lab.github.io/monocle3/

**Other**		

Anti-FLAG® M2 Magnetic Beads	Sigma-Aldrich	Cat# M8823
GFP-Trap® Magnetic Particles M-270	ChromoTek	Cat# gtd20; LOT: LM0000172
Zeiss Celldiscoverer 7	Zeiss	N/A
Zeiss LSM-700 confocal microscope	Zeiss	N/A
Olympus IX2-UCB immunofluorescent microscope	Olympus	N/A
QuantStudio 3 Real-Time PCR System	Thermo Fisher Scientific	N/A
C1000 Touch Thermal Cycler	Bio-Rad	N/A
Biotek Synergy HTX plate reader	Agilent Technologies	N/A

### Experimental model and study participant details

#### Human placental tissue collection

Human placental tissue from weeks 5–12 gestational age was obtained from elective pregnancy terminations with informed patient consent according to methods approved by the University of Alberta Human Research Ethics Board (Pro00089293). All placental samples and patient characteristics for samples in this study can be found in Table S1.

Biological sex was determined for 14 placental samples; 57.14% were male, 42.86% were female.

#### Explant cultures

Human placental tissue was obtained with informed consent from individuals undergoing elective terminations.

##### ST-intact explants

Placental tissue was washed with cold PBS then cut into tissue pieces (∼2mm^3^). Explants were cultured one/well, triplicate per treatment in floating explant medium [Iscove’s Modified Dulbecco’s Medium (IMDM; Gibco, 12440061) supplemented with 10% fetal bovine serum (FBS; Wisent, Multicell Inc., Lot: 185730), 100U/mL Penicillin and 100μg/mL Streptomycin (Gibco, 15140122)] as per Patel et al. (2023) in humidified incubators with 5% CO_2_ and atmospheric O_2_. After 24 h, explants were treated with 5 μM myristoylated aPKC pseudosubstrate inhibitor (Invitrogen, 77749) or solvent controls. After an additional 24 h of culture, explants were fixed with 4% PFA for immunofluorescence.

##### ST regeneration explants

Tissue pieces (∼2mm^3^) were denuded of ST and cultured as per Duan et al. (2025) Briefly, placental tissue was washed with cold PBS cut into explants, then trypsinized (0.25% trypsin-EDTA, Gibco, 25200-056). Explants were cultured one/well, triplicate per treatment in explant regeneration medium [IMDM supplemented with 10% FBS, 1% ITS-X (Gibco, 51500-056), and 50μg/mL gentamycin (Gibco, 15750-060)] in humidified incubators with 5% CO_2_ and atmospheric O_2_. After 24 h, the tissue was vigorously washed to remove ST, debris, and treated with *PRKCZ* – targeting siRNA KD (Dharmacon, J-003526-14), non-targeting control siRNA (Dharmacon, D-001810-10), 5 μM myristoylated aPKC pseudosubstrate inhibitor, or 3 μM LATS inhibitor (TDI-011536; MedChemExpress, HY-150042). After an additional 48 h of culture, explants were fixed with 4% PFA for immunofluorescence staining or collected for western blotting.

#### Primary pCT *in vitro* ST differentiation

Primary human first trimester pCTs were isolated and cultured as previously reported (Guilbert et al., 2002; Patel et al., 2023; Shaha et al., 2022). For primary *in vitro* 72 h ST differentiation, pCTs were cultured in IMDM supplemented with 10% FBS and penicillin–streptomycin in a 5% CO_2_ as per Shaha et al. (2022). Cells were seeded for 4 h, then washed and treated with 10 μM 8-Br cAMP (Sigma-Aldrich, B7880) in IMDM +10% FBS and pen-strep overnight. The following morning, medium was changed for 8-Br-cAMP removal, and the cells were cultured for an additional 48 h before fixation.

#### Human trophoblast stem cell culture

The human trophoblast stem cell lines (Okae et al., 2018) (TSC; CT27, CT29) were obtained from Riken Biosource Resource Center and maintained on 5 μg/mL collagen IV (Corning, 354233) coated plates and cultured with human TSC culture medium (Duan et al., 2025; Okae et al., 2018) in a humidified incubator at 37°C with 5% CO_2_ and atmospheric O_2_. Human TSC lines were used from passage 20–29 and split at 1:5–1:20 ratios.

#### Human trophoblast organoid culture

TSC CT27 (Female line) and CT29 (Male line) cells were passaged and cultured in High Aspect Ratio Vessels (HARVs) (Synthecon) using a Rotary Cell Culture System (Synthecon). Organoids were pelleted via centrifugation, washed with PBS, and prepared for downstream analyses. For single nuclei RNA-sequencing, PBS was removed and the organoids were flash frozen. For western blotting, organoids were lysed in RIPA buffer. For immunofluorescent staining, organoids were fixed in 4% PFA for 10 min in siliconized 2mL round bottom tubes (Sigma, SL2).

#### BeWo cell line maintenance

BeWo cells were maintained in Ham’s F-12 Nutrient Mix (Gibco, 11765047) supplemented with 15% FBS and Penicillin-Streptomycin (100U/mL Penicillin, 100μg/mL Streptomycin) in a humidified incubator at 37°C with 5% CO_2_ and atmospheric O_2_.

#### BeWo *in vitro* ST differentiation

To induce *in vitro* ST differentiation, BeWo cells were seeded on glass coverslips coated with 0.2% gelatin (Sigma, G1393). Medium was changed to also include 500μM 8-Br-cAMP, and refreshed every 48 h. Cells were fixed after a total of 96 h of 8-Br-cAMP treatment. For western blotting assessment of p-YAP(Ser127) and *p*-LATS1(Ser909) expression, BeWo cells were seeded at 15% confluency, then transfected with aPKC-ζ III-FLAG the next day using Lipofectamine LTX reagent with PLUS reagent (Invitrogen, 15338100) in Opti-MEM I Reduced Serum Medium (Gibco, 31985062) according to manufacturer’s protocols. After 24 h, cells were collected at 0 and 2 h post 8-Br-cAMP treatment in RIPA supplemented with Protease Inhibitor Cocktail (Sigma-Aldrich, P2714) and Halt Phosphatase Inhibitor Cocktail.

#### HEK293T cell line culture

HEK293T were maintained in DMEM F12 (Gibco, 11320033) supplemented with 10% FBS and Penicillin-Streptomycin (100U/mL Penicillin, 100μg/mL Streptomycin) in a humidified incubator at 37°C with 5% CO_2_ and atmospheric O_2_. HEK293T cell line was used from passage 3–10 and split at 1:10–1:30 ratios.

### Method details

#### Single nuclei RNA sequencing

10× Genomics snRNA-seq techniques were used for flash frozen organoids, and we adapted the single nuclei sequencing data from first trimester placentas previously presented by Wang et al. (2024).

Libraries were prepared by sequencing flash-frozen placental organoids by using a Chromium Nuclei Isolation with RNAse Inhibitor kit (Novogene, 1000494). Partial lane dual index sequencing was performed by the Princess Margaret Genomics Center (PMGC) via Illumina next generation sequencing (Illumina NovaSeqX) for a total of 200M read pairs per sample. The sequenced data has been deposited in GEO under accession number (GSE310653). The first trimester single cell nuclei dataset was adapted from the previously published sequencing data (Wang et al., 2024).

Sample demultiplexing, gene counting, and feature barcode analysis was performed on CellRanger-9.0.1. (Zheng et al., 2017). Downstream analysis was performed in R-4.5.1 and Seurat −5.3.0. (Hao et al., 2024). Both datasets were filtered by excluding samples with less than 200 or more than 2500 features, as well as samples with more than 20% of mitochondrial counts. Dimension reduction was performed via UMAP, and the cell clusters were identified utilizing canonical markers for the cell identities.

Filtering was performed in both samples, and we retained a total of 45,697 nuclei for the first trimester dataset, and 22,250 nuclei for our organoid dataset. Batch correction and integration were performed in both datasets. The datasets were visualized using UMAP dimensional reduction analysis. For both datasets, clusters were annotated into the appropriate cell type by analyzing the expression of different canonical cell markers previously described (Figures S1 and S2) (Duan et al., 2025; Keenen et al., 2025). The pseudotime analysis was performed using the R package Monocle3 (Cao et al., 2019). Trajectory reconstruction was performed in both the first trimester and the organoids dataset, with the bipotential pCTs population in the former and the pCT population in the latter used as the root for trajectory inference.

#### Bulk RNA sequencing

Libraries were prepared using the Illumina Stranded mRNA prep kit. Partial lane sequencing was performed by the Genome Science Center (GSC) at the University of British Columbia via Illumina next generation sequencing (NovaSeq X Plus Series PE150) for 100M read pairs per sample. Sequencing data have been deposited in GEO under accession number (GSE310653).

Quality control and library alignment of the samples was performed in Ubuntu via the FastQC and STAR distros, respectively (Dobin et al., 2013). We utilized R for the downstream analysis of the data. Counts were obtained utilizing the Rsubread package (Liao et al., 2019). Analysis of the data, estimation of fold changes and dispersion was performed using DESeq2 (Love et al., 2014). The GO pathways were generated using the clusterprofiler package (Wu et al., 2021).

#### *PRKCZ* knockdown organoids

*PRKCZ* – targeting siRNA KD or non-targeting controls were transfected into TSC using Opti-MEM I Reduced Serum Medium (Gibco; cat# 31985062) and Lipofectamine LTX Reagent with PLUS Reagent (Invitrogen; cat#15338100) according to manufacturer’s recommendations. 24h post transfection, treated TSCs were moved into HARVs for 24–48 h in rotational culture.

#### Generation of CRISPR-Cas9 *PRKCZ* knockout cells

The Alt-R CRISPR-Cas9 System was used to create *PRKCZ* KO lines. *PRKCZ* targeting guide RNAs (CRISPR RNA; crRNA) were designed using the IDT Alt-R CRISPR HDR Design Tool to target base pairs 100584–100603 (tccagta gacgacaaga a) on the *PRKCZ* gene. Control lines were created using the Alt-2 Cas9 Neg Ctrl crRNA #1 (IDT, cat# 1072544, lot#0000828510). A two-part guide RNA, crRNA and Alt-R CRISPR Cas9 tracrRNA-ATTO 550 (tracrRNA; IDT, cat# 1075927, lot: 00005558022) were combined in equimolar concentrations for a final duplex concentration of 1 μM in Nuclease-Free Duplex Buffer (IDT, cat#11-01-03-01). Ribonucleoprotein (RNP) complexes were transfected with Lipofectamine CRISPRMAX Transfection Reagent (Invitrogen, Cat# CMAX00001, lot 2634820) according to manufacturer’s protocols. Briefly, Alt-R S.p. Cas9 nuclease V3 (IDT, cat#1081058, lot 0000833182) and crRNA/tracrRNA duplex were transfected using the Cas9 Plus Reagent (Invitrogen, Cat# 100035624, lot 2634802) and Lipofectamine CRISPRMAX Reagent (Cat# 100035629, lot 2650857) in Opti-MEM I Reduced Serum Medium (Gibco, 31985062). 24 h later, cells were sorted via Fluorescence-Activated Cell Sorting Flow Cytometry for ATTO 550.

#### BeWo siRNA knockdown

To perform *PARD3* knockdowns, BeWo cells were seeded on glass coverslips coated with 0.2% gelatin. After 24 h, *PARD3-*targeting siRNA [ON-TARGETplus Human PARD3 (56288) siRNA; (Dharmacon J-015602-06)] and non-targeting controls were transfected into BeWos using Opti-MEM I Reduced Serum Medium and Lipofectamine LTX Reagent with PLUS Reagent in Opti-MEM I Reduced Serum Medium according to manufacturer’s recommendations. The following day, *in vitro* ST differentiation was induced as above.

#### Immunoprecipitations

For Par-3-EGFP and aPKC-ζ III-FLAG immunoprecipitations, HEK293T cells were seeded at 30% density and transfected the following day with 1:1 plasmid ratio using Lipofectamine LTX Reagent with PLUS Reagent according to manufacturer’s recommendations. Cells were lysed with ice-cold lysis buffer [50 mM Tris–HCl pH 7.4, 1% IGEPAL, 150 mM NaCl, and 1:100 Protease inhibitor (P2714,Sigma-Aldrich, St. Louis, MO, USA)], incubated for 15 min with end-over-end rotation at 4°C, and centrifuged for 15 min at 14,000 RCF. Protein assays were performed using Pierce BCA Assay Kit (Thermofisher, 23227). 50μL of Anti-FLAG M2 Magnetic Beads (Sigma-Aldrich, M8823) were incubated for 4 h at 4°C with 500μg protein lysate. Par-3-EGFP, LATS1-Myc, and aPKC-ζ III-FLAG immunoprecipitations HEK293T cells were seeded at 10% density and transfected the following day plasmids for Par-3-EGFP IPs with LATS1-Myc and aPKC-ζ III-FLAG. Cells were lysed with ice-cold lysis buffer modified from Lv et al. (2015). [50 mM Tris–HCl pH 7.5, 0.3% IGEPAL, 150mM NaCl, 1mM EDTA-disodium, 1:100 Protease inhibitor, and 1:100 Phosphatase inhibitor (Halt Phosphatase Inhibitor Cocktail, Thermo Scientific, 78420), then incubated for 30 min with end-over-end rotation at 4°C and centrifuged for 30 min at 14,000 RCF. 25μL of GFP-Trap Magnetic Particles M-270 (ChromoTek, gtd20; LOT; LM0000172) were incubated overnight at 4°C with 500μg protein lysate. Protein was eluted by boiling with 1× SDS buffer.

#### Live cell imaging

HEK293T cells transfected with Par-3-EGFP or aPKC-ζ III-EGFP, and human TSC transfected with aPKC-ζ III-EGFP were analyzed at 24 h. Images were captured on a Zeiss Celldiscoverer 7 with an Axiocam 712 mono camera and Zeiss Plan-Apochromat 20×/0.7 autocorr lens.

#### Plasmids

aPKC-ζ III-FLAG and aPKC-ζ III-EGFP plasmids were constructed and packaged by VectorBuilder. Vector IDs can be used to retrieve detailed information about the vector on vectorbuilder.com. pEGFP-N1-Par3 plasmid was a gift from Dr. Masanori Nakayama. pcDNA3 Lats1 (Nigg HS189) (LATS1-Myc Tag) was a gift from Erich Nigg (Addgene plasmid # 41156; http://n2t.net/addgene:41156; RRID:Addgene_41156) (Chan et al., 2005).

#### Immunofluorescence staining

##### Placental tissue

Placental explants and tissue were simultaneously permeabilized and blocked using blocking buffer [5% Normal donkey serum, 0.5% Triton X-100, and 1:100 human IgG (Invitrogen, 02–7102)], then incubated overnight with primary antibodies. For Ki67 staining, sodium citrate antigen retrieval was performed, then explants were blocked as above. The following day, tissue was washed and incubated with secondary antibodies and/or Phalloidin and Hoechst 33352. Tissue was mounted using imaging spacers and Fluoromount-G (SouthernBiotech, 0100-01).

##### Human trophoblast organoids and 2D cells

Human trophoblast organoids were stained as previously described (Duan et al., 2025). Briefly, organoids or cells (BeWo and primary *in vitro* differentiated ST) were permeabilized, blocked in blocking buffer (5% NDS, 0.01% Tween 20, and 1:100 human IgG), then incubated with primary antibodies overnight. The next day, they were washed then incubated with secondary antibodies and/or Phalloidin and Hoechst 33352. Organoids were mounted using imaging spacers and Fluoromount-G. Cells cultured on coverslips were mounted with Fluoromount-G.

#### Image capture and analysis

Confocal microscopy was used to capture explant and organoid images. Three regions per explant or five organoids per treatment were captured using a Zeiss Plan Apochromat-20×/0.8 M27 or Zeiss Plan Apochromat-63×/1.4 M27 oil lens on a Zeiss LSM-700 confocal microscope. Z-stacks of placental explants(15-55μm) were captured at 20× (2.02μm step size) and 63× (1.2 μm step size) (30-60μm). Z-stacks of organoids (20-30μm) were captured at 20× (2.02μm step size). XY-images were captured at 20×. Images were analyzed using Volocity Imaging Software (Quorum Technologies, version 7.0.0). For 2D cell fusion assessment, triplicate images per treatment were captured at 10× magnification using an Olympus IX2-UCB immunofluorescent microscope equipped with a Roper Scientific camera and aa Sutter Instruments Lambda DG-4 fluorescent lamp and cellSens Dimensions imaging software.

#### Fusion assessment

##### Explants

ST regeneration of placental explants was quantified as previously described.(Duan et al., 2025) Briefly, explants stained for E-cadherin (pCT marker), Phalloidin (F-actin), and nuclei were used to assess fusion. Single 20× XY-plane cross sectional images were assessed. Single nuclei surrounded by E-cadherin were considered pCT, and multiple nuclei surrounded by phalloidin and E-cadherin signal were considered ST. For each treatment, the area of ST/Area of pCT was normalized to donor-matched 24 h control trypsinized samples, then to regenerated controls.

##### Cells

Images were blinded for treatments and the number of nuclei incorporated into multinucleated E-cadherin positive clusters were counted and divided by the total number of nuclei using ImageJ and Volocity Imaging Software.

#### Western blotting

Protein was collected using RIPA (150 mM NaCl, 1% Triton X-100, 0.1% SDS, 50 mM Tris, and 0.5% Sodium deoxycholate) supplemented with Protease Inhibitor Cocktail (Sigma-Aldrich, P2714) and Halt Phosphatase Inhibitor Cocktail (Thermoscientific, 78420) for phospho-specific antibody detection. SDS-PAGE was run using 10-20μg protein. Membranes were blocked with 0.3% skim milk powder and incubated overnight with primary antibodies. The following day, membranes were washed then incubated with secondary antibodies. After incubation with phospho-specific antibodies, membranes were stripped for 4 × 30 min (7.5g glycine, 0.5g SDS, 5mL Tween 20, pH 2.2 w HCl), washed, blocked, and re-incubated with non-phosphorylated antibodies overnight. Total protein was determined using Fast Green stain (0.001% Fast Green FCF (w/v), 30% methanol, 7% acetic acid), then destained (30% methanol, 10% acetic acid). Membranes were imaged using the Licor Odyssey CLx and analyzed using Image Studio (V5.5).

#### RNA isolation and RT-PCR

Organoids were harvested and RNA was extracted using TRIzol-chloroform extraction and purified using PureLink RNA Mini Kit (Invitrogen). Reverse transcription was performed using iScript cDNA Synthesis Kit (BioRad) with 1000ng RNA, and cDNA was diluted 1:10 for all reactions. RT-PCR reactions were performed using SYBR Green Universal Master Mix (Applied Biosystems) on a QuantStudio 3 Real-Time PCR System (Thermo Fisher Scientific) and analyzed using QuantStudio Design & Analysis Software (Thermo Fisher Scientific). The 2^−ΔΔCT^ method was used to calculate relative change in mRNA expression using housekeeping gene *RNA18SN1* (Jaremek et al., 2023; Livak and Schmittgen, 2001).

*SRY* expression was used to determine the biological sex of placental samples. The amplicon was generated by performing PCR using PCR Supermix (Invitrogen) (amplicon = 169 bp) run on a C1000 Touch Thermal Cycler (Biorad, 1851148). Gel electrophoresis was performed to detect amplicons and visualized using RedSafe Nucleic Acid Staining Solution (Froggabio).

#### ELISA

β-hCG ELISAs were performed on medium collected from 24 h control and *PRKCZ* KD organoids using a β-hCG ELISA kit (EIA-1911; DRG International). Plates were analyzed using a Biotek Synergy HTX plate reader (Gen 5 Software). Β-hCG concentration was normalized to total protein of of samples.

### Quantification and statistical analysis

#### vCT proliferation quantification

Proliferation in placental explants was quantified by staining explants for Ki67 (proliferation marker), E-cadherin (pCT marker), and nuclei (Hoechst). The percentage of Ki67 positive pCTs were determined by counting the number of Ki67 positive nuclei surrounded by E-cadherin junctions (pCT marker), then dividing by the total number of nuclei surrounded by E-cadherin junctions.

$$\%\;Ki67\;pCTs=\frac{\left(\#Ki67\;Positive\;Nuclei\;within\;E-cadherin\;Junctions\right)}{Total\;\#\;Nuclei\;within\;E-cadheirn\;Junctions\;}X100$$

#### Fusion quantification

A multinucleated cell was determined by the breakdown of E-cadherin junction between two or more nuclei. Percent Fusion was calculated by the ratio of the number of nuclei incorporated into multinucleated cells by the total number of nuclei.

$$\%\;Fusion=\frac{\#Nuclei\;Incorporated\;into\;Multinucleated\;Cells}{Total\;\#\;Nuclei}X\;100$$

Relative fusion was determined by the ratio of the percent fusion of the treatment:control.

$$Relative\;Fusion=\frac{\%\;Fusion\;of\;Treatment\;Group}{\%\;Fusion\;of\;Control\;Group}$$

Fusion index was calculated by the subtracting the number of nuclei in multinucleated cells by the total number of multinucleated cells, then dividing by the total number of nuclei.

$$Fusion\;Index=\frac{\left(\#\;Nuclei\;Incorporated\;into\;Multinucleated\;Cells\right)-\left(\#\;Multinucleated\;Cells\right)}{Total\;\#\;Nuclei}$$

### Statistical analysis

All statistical analyses were performed in GraphPad PRISM (Version 10.4.2). For all tests, the threshold for significance is *p* > 0.05. Statistical tests used for each experimental design are stated within figure legends. All graphs and representative images from different placental tissue donors are from at least three biological replicates or cell lines with at least three experimental replicates.

## Supplemental information

Document S1. Figures S1–S10 and Tables S1

Document S2. Article plus supplemental information
