# Supplementary material for: aPKC-ζ III promotes trophoblast fusion by altering Par-3 interactions with Hippo signaling kinase LATS1
Source: Stem Cell Reports. 2026 Jun 25;21(7):102975. doi: 10.1016/j.stemcr.2026.102975 (PMC13385426; doi:10.1016/j.stemcr.2026.102975)
Supplement: Document S1. Figures S1–S10 and Tables S1 [file mmc1.pdf]

Stem Cell Reports, Volume *21*

Supplemental Information

aPKC-ζ III promotes trophoblast fusion by altering Par-3 interactions with Hippo signaling kinase LATS1

Sumaiyah Z. Shaha, Wendy K. Duan, Juan Garcia Rivas, Ivan K. Domingo, and Meghan Riddell

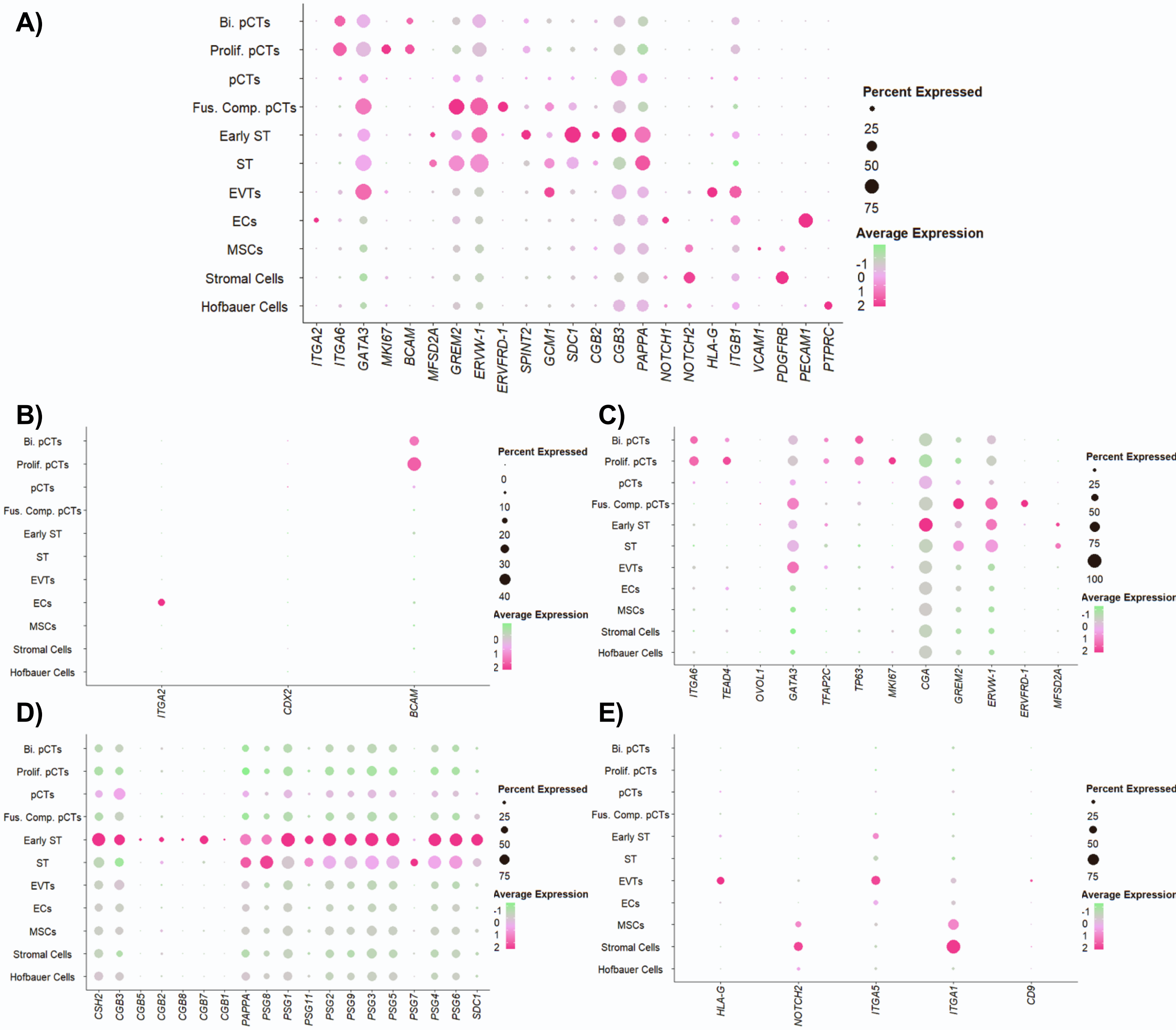

Supplementary Figure 1: Subcluster defining marker gene expression in first trimester placenta. Dot plots highlighting A) key marker genes, B) TSC, C) pCT, D) ST, and E) EVTs. PCTs = progenitor cytotrophoblasts; Bi. pCTs = bipotential pCTs; Prolif. pCTs = proliferative pCTs; Fus. Comp. pCTs = fusion competent pCTs; ST = syncytiotrophoblast; EVTs = extravillous trophoblast; ECs = endothelial cells; MSCs = mesenchymal stem cells.

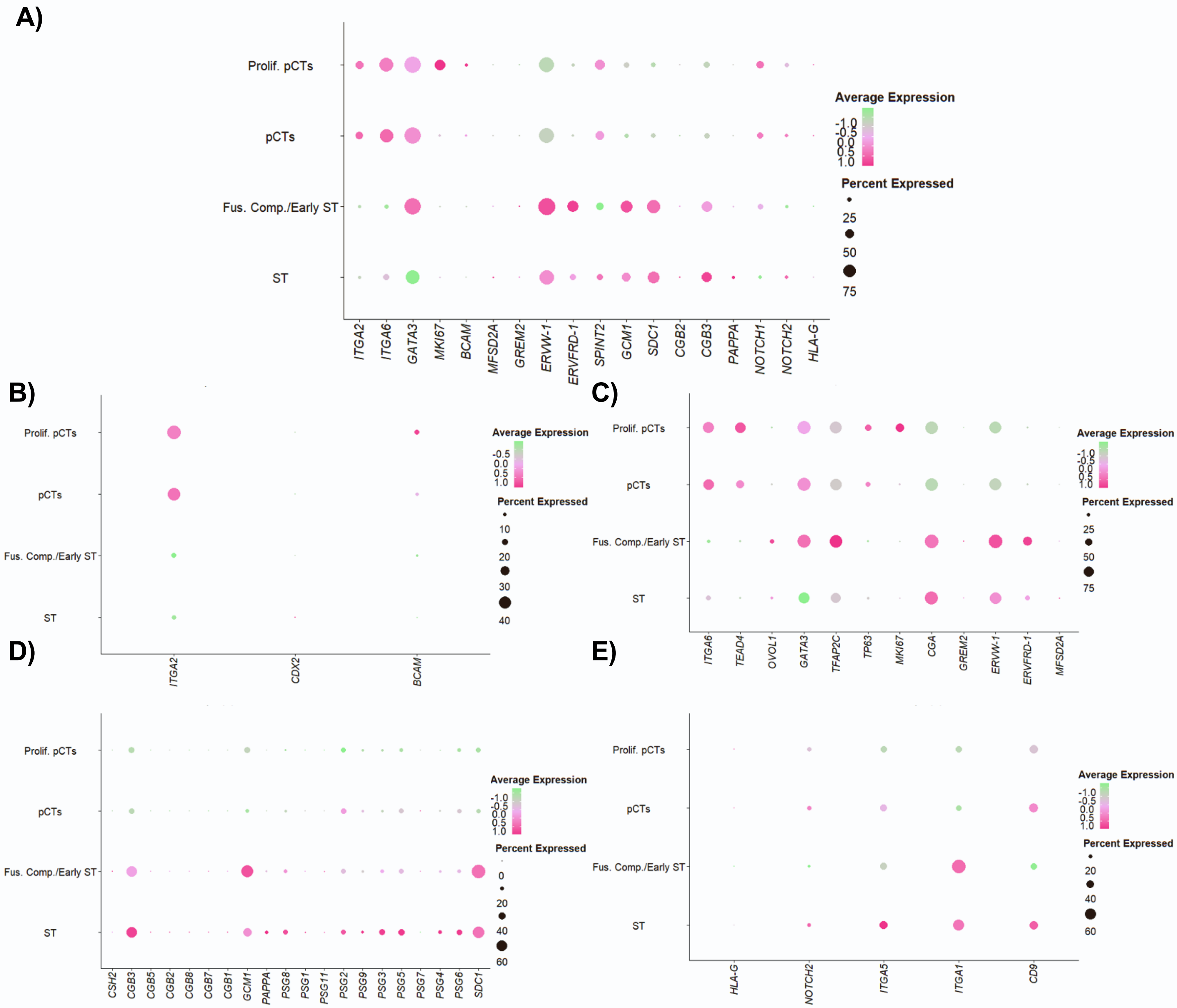

Supplementary Figure 2: Subcluster defining marker gene expression in TSC organoids. Dot plots highlighting key marker genes in A) all cell types, B) TSC, C) pCT, D) ST, and E) EVTs. PCTs = progenitor cytotrophoblasts; Prolif. pCTs = proliferative pCTs; ST = syncytiotrophoblast; Fus. Comp. pCTs / early ST= fusion competent pCTs / early ST.

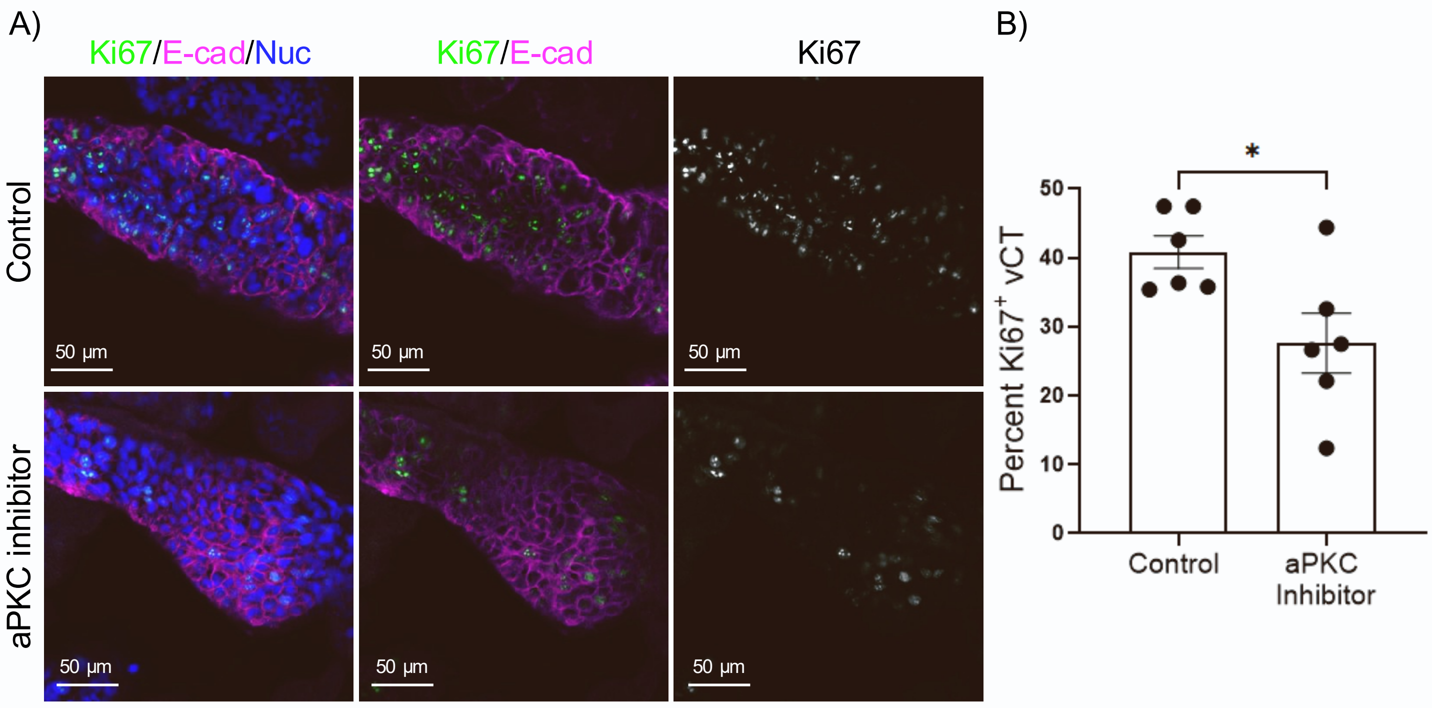

Supplemental Figure 3. A) Representative XY plane confocal microscopy images of 24 hour control and aPKC inhibitor treated first trimester placental explants stained for Ki67 (green), E-cadherin (E-cad; magenta), and Nuclei (Nuc; blue); scale bars = 50µm. B) Summary data for the percent of Ki67 positive villous cytotrophoblasts (vCT); unpaired Students T-test; Data are from n=6 placentas; Graphs are mean +/- S.E.M,; *p ≤ 0.05.

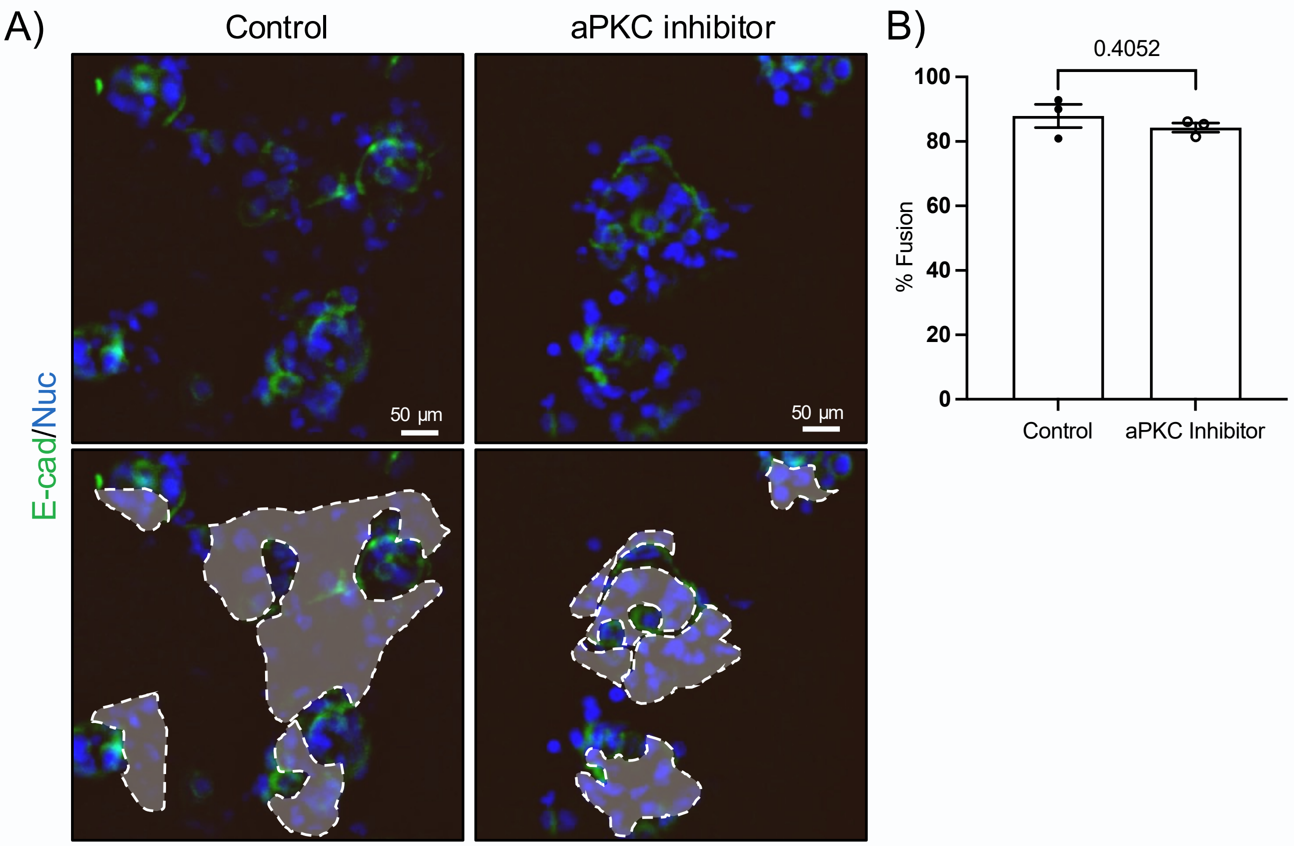

Supplementary Figure 4. A) Representative images of control and aPKC inhibitor treated primary first trimester *in vitro* differentiated ST stained for E-cad (E-cadherin; green) and nuclei; dashed regions below indicate regions of multinucleated cells. B) Summary data of percent fusion in control and aPKC inhibitor treated cells; Data are mean +/- S.E.M., paired t-test, Data are from *n*=3 placentas.

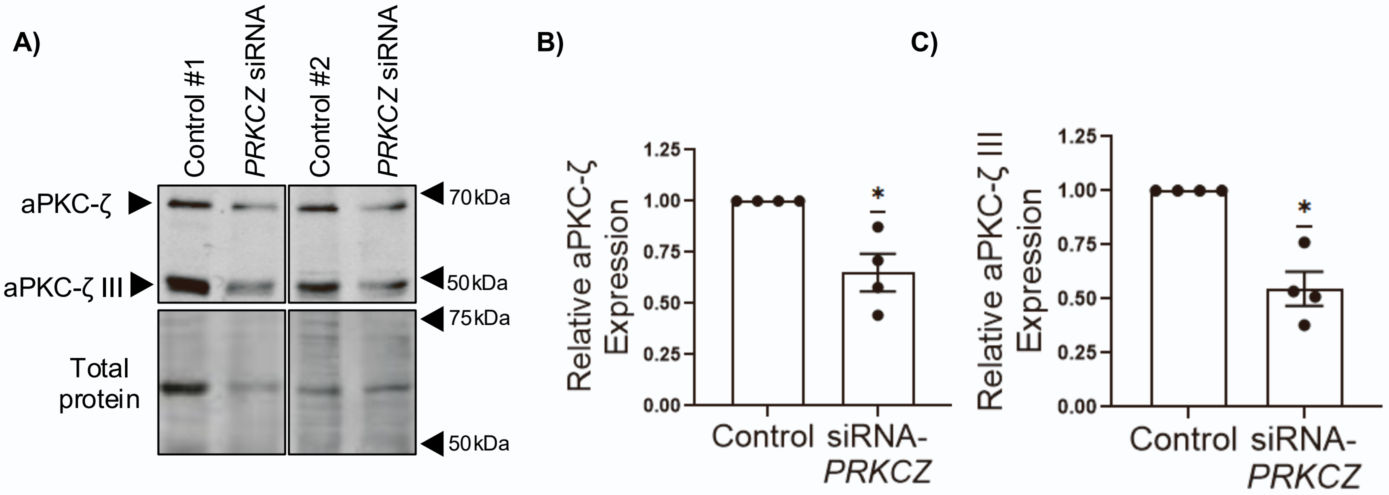

Supplementary Figure 5: Knockdown of aPKC-ζ isoforms in placental explants A) Representative western blot of aPKC-ζ and aPKC-ζ III in placental explants treated +/- *PRKCZ*-targeting siRNA;Summary data of relative B) aPKC-ζ andC) aPKC-ζ III expression; Data are mean +/- S.E.M., one sample t-test, *p ≤ 0.05, Data are from *n*=4 placentas.

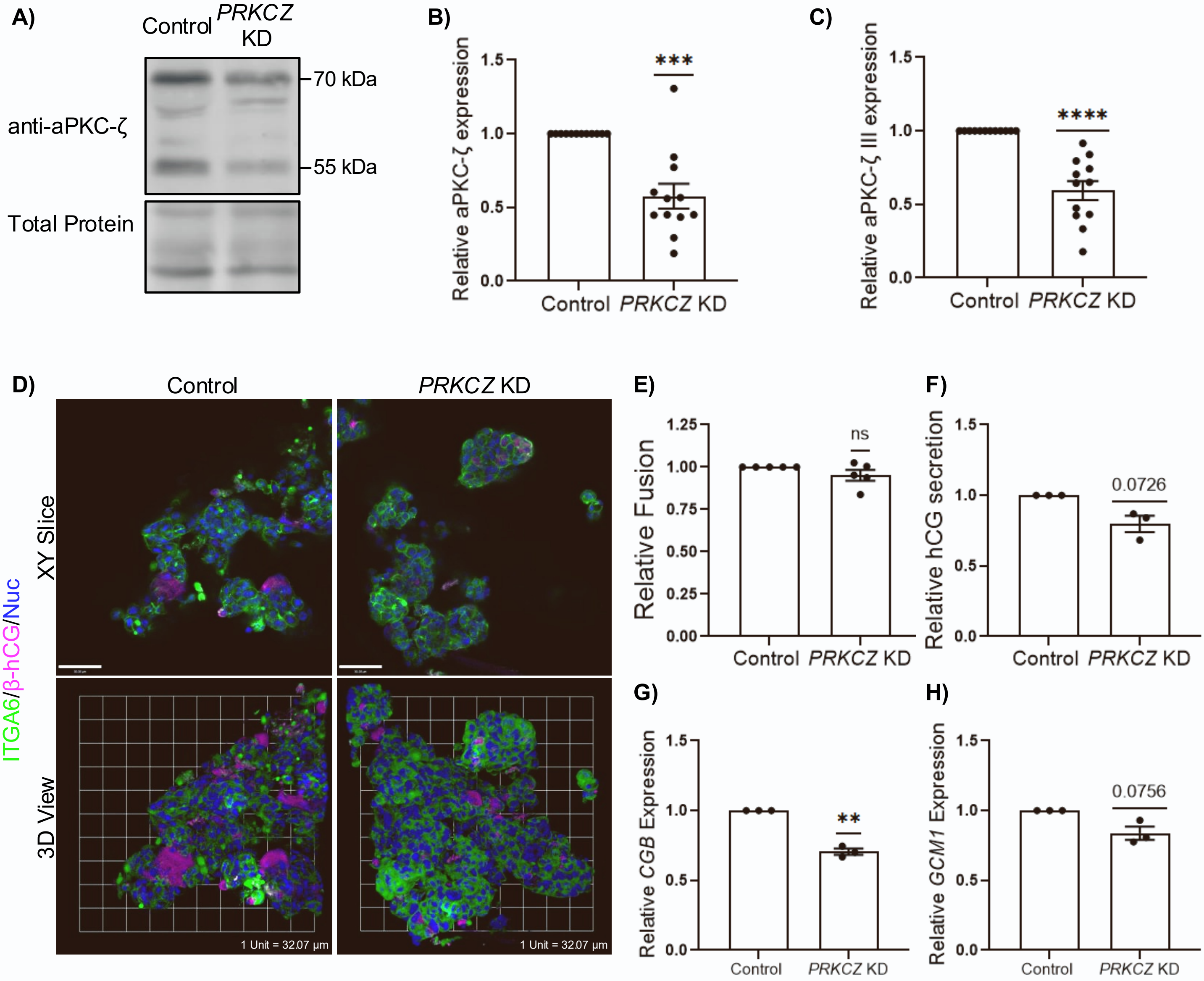

Supplementary Figure 6: A) Representative western blot of aPKC-ζ expression in control and *PRKCZ* KD undifferentiated TSC. Summary data of relative B) aPKC-ζ and C) aPKC-ζ III protein expression; one sample t-test, Data are from *n*=11-12 individual experiments. D) Representative XY plane (top) and 3D reconstituted (bottom) confocal microscopy images of control and *PRKCZ* KD trophoblast organoids stained for ITGA6 (green), β-hCG (magenta), and nuclei (blue); scale bars = 50µm. E) Summary data for relative fusion of control and *PRKCZ* KD organoids;, one sample t-test, Data are from *n*=5 individual experiments. F) Relative β-hCG secretion in control and *PRKCZ* KD organoids; Relative expression of G) *CGB* and H) *GCM1* in control and *PRKCZ* KD organoids; one sample t-test, Data are from *n*=3 individual experiments. Graphs for all data are mean +/- S.E.M,; *p ≤ 0.05, **p ≤ 0.01 ***p ≤ 0.001, ****p ≤ 0.0001.

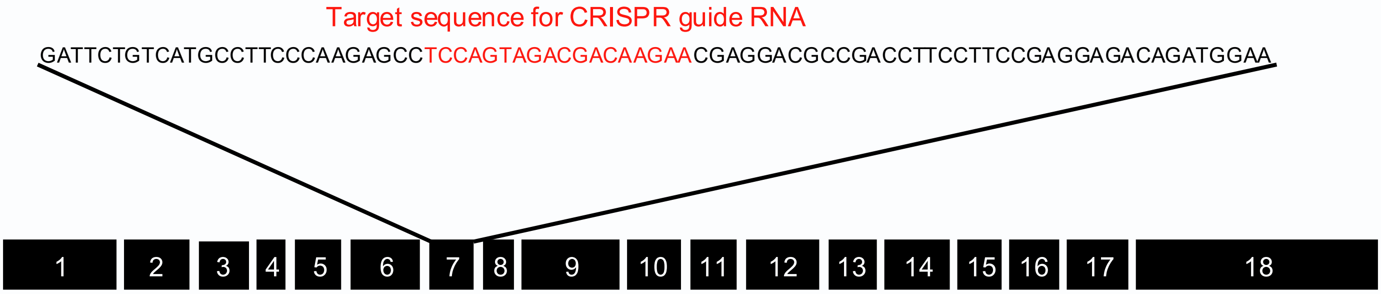

Supplementary Figure 7: Schematic diagram of the *PRKCZ* gene with the 18 exons encoding for the full length aPKC-ζ. Guide RNA target (Red highlighted sequence) for CRISPR Cas9 mediated knockout of *PRKCZ.* Exons 7-18 are conserved in aPKC-ζ III.

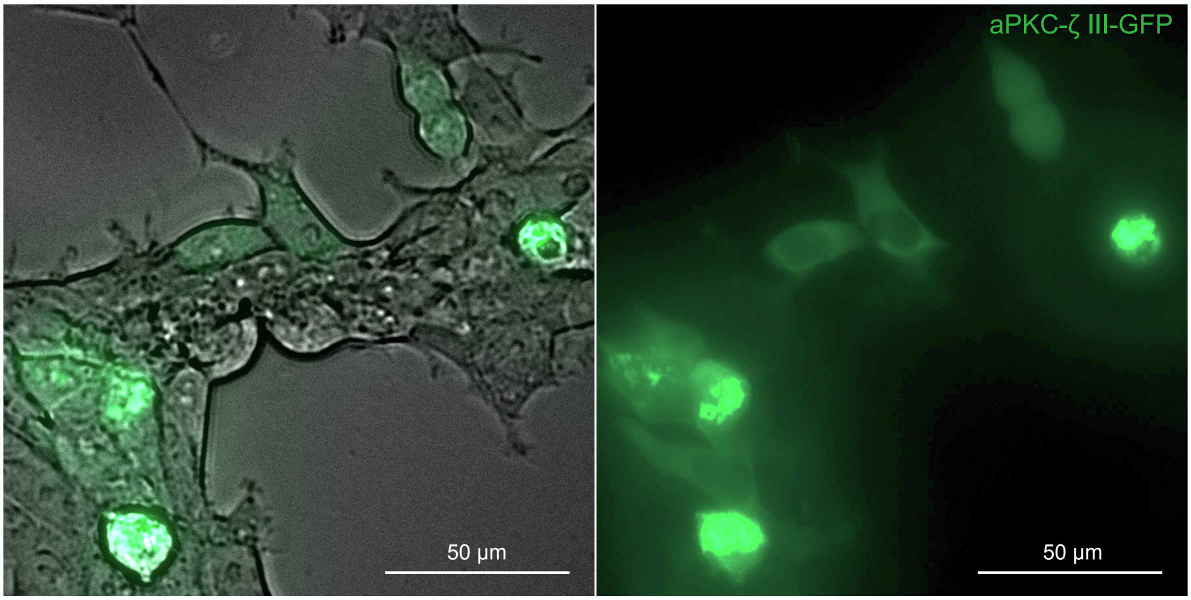

Supplementary Figure 8: Live cell imaging of HEK293T cells transfected with aPKC-ζ III-EGFP plasmid.

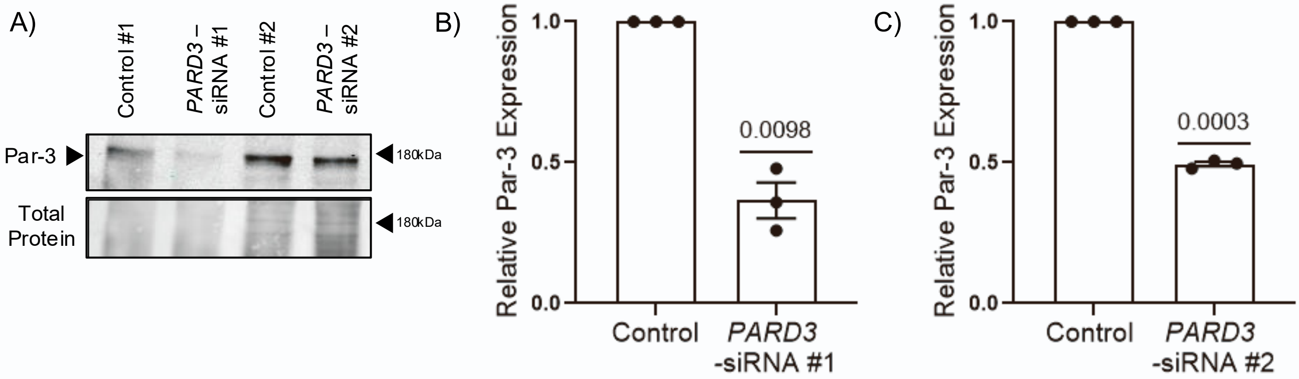

Supplementary Figure 9: A) Representative western blot of Par-3 expression in BeWo cells treated with *PARD3*-targeting siRNA; Summary data for relative Par-3 expression of B) *PARD3*-targeting siRNA #1 and C) *PARD3*-targeting siRNA #2; Data are mean +/- S.E.M., one sample t-test, *n*=3; *p ≤ 0.05, ***p ≤ 0.001, ****p ≤ 0.0001; Data are from n=3 individual experiments.

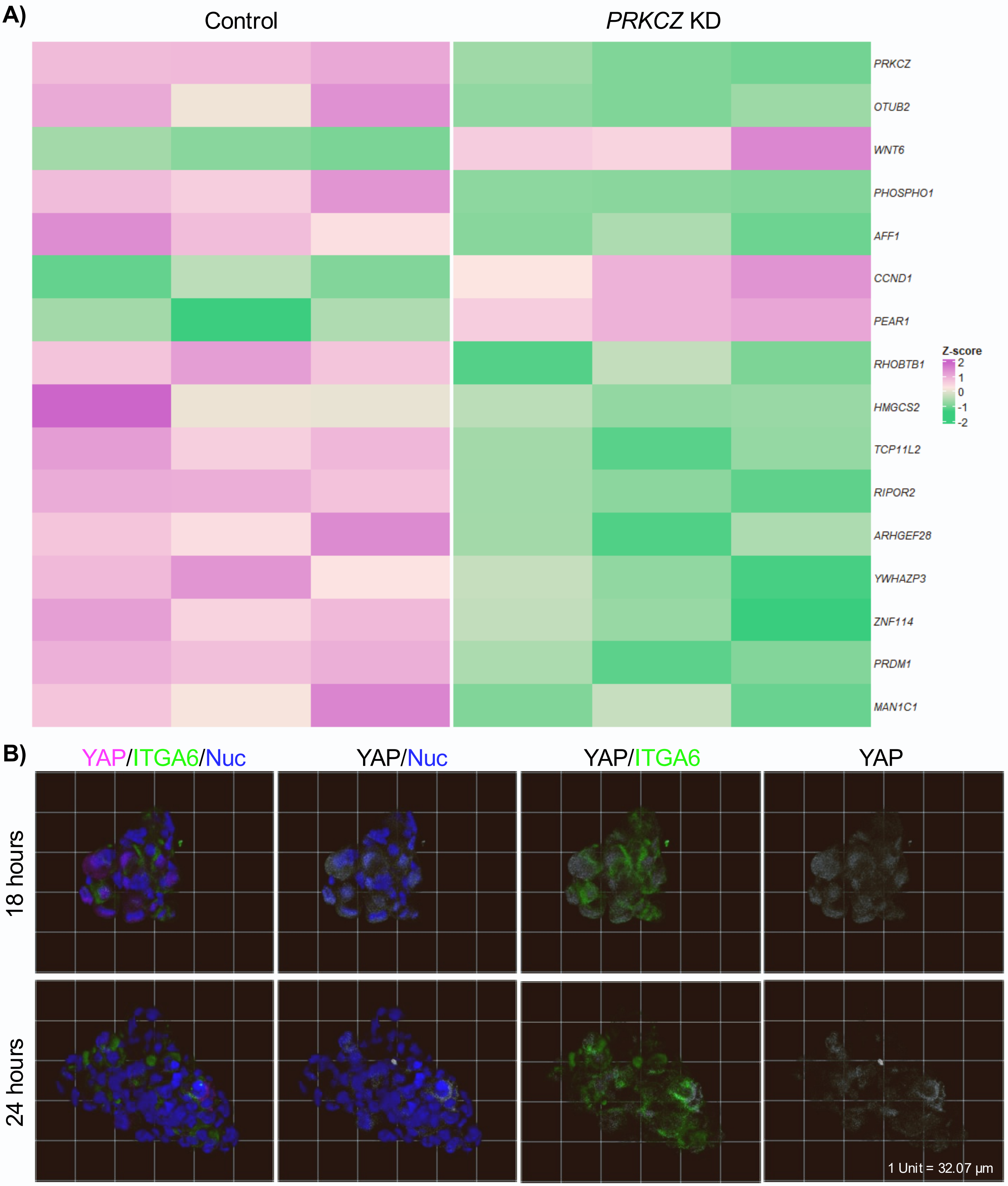

Supplementary Figure 10: A) Heatmap of significant differentially expressed genes in control and *PRKCZ* KD trophoblast organoids identified by bulk-RNA seq.; Data are from n=3 individual experiments per control and *PRKCZ* KD group B) Representative 3D reconstituted confocal microscopy images of 18 and 24 hour trophoblast organoids stained for YAP (magenta), ITGA6 (green), and nuclei (blue).

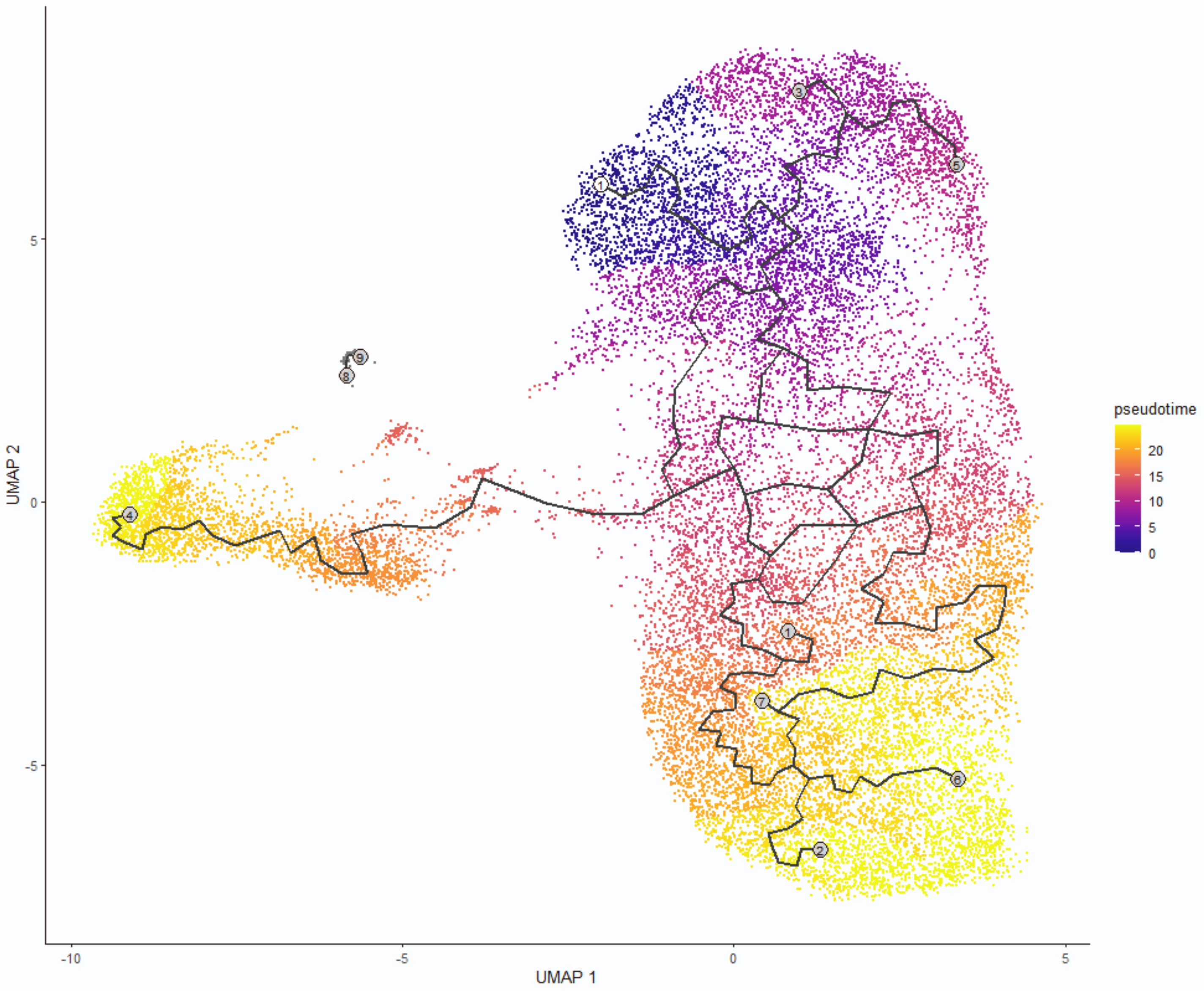

Supplementary Figure 11: Pseudotime trajectory analysis of trophoblast organoids.

Supplementary Table 1. Sample and patient characteristics

	Placental Characteristics		Maternal Characteristics	
	Mean	S.D.	Mean	S.D.
Age (mean+/- S.D.)	8.9	2.096064973	26.75757576	6.260143284
Range (min, max)	(5, 12.7)		(18, 41)	
Count	44		33	

Biological sex was determined for *PRKCZ* KD and LATS inhib. placental explant experiments; Data are from n=6 placentas.
